# Quantifying Magnetic Anisotropy of Series of Five‐Coordinate Co^II^ Ions: Experimental and Theoretical Insights

**DOI:** 10.1002/advs.202415624

**Published:** 2025-01-14

**Authors:** Vijaya Thangaraj, Daniele Sartini, Dipanti Borah, Deepanshu Chauhan, Vasudha Sharma, Lorenzo Sorace, Gopalan Rajaraman, Mauro Perfetti, Maheswaran Shanmugam

**Affiliations:** ^1^ Department of Chemistry Indian Institute of Technology Bombay Powai Mumbai Maharashtra 400076 India; ^2^ Dipartimento di Chimica “Ugo Schiff” and UdR INSTM Università degli Studi di Firenze Via della Lastruccia 3−13 Sesto Fiorentino 50019 Italy

**Keywords:** ab initio calculations, cobalt, coordination chemistry, EPR, torque magnetometry

## Abstract

Stabilizing large easy‐axis type magnetic anisotropy in molecular complexes is a challenging task, yet it is crucial for the development of information storage devices and applications in molecular spintronics. Achieving this requires a deep understanding of electronic structure and the relationships between structure and properties to develop magneto‐structural correlations that are currently unexplored in the literature. Herein, a series of five‐coordinate distorted square pyramidal Co^II^ complexes [Co(L)(X_2_)].CHCl_3_ (where X = Cl (**1**), Br (**2**), or I (**3**)) is reported, all exhibiting easy‐axis magnetic anicotropy. The size of the zero field splitting axial parameter (*D*) is quantitatively determined (**1** = −72; **2** = −67 and **3** = −25 cm^−1^) using a cantilever torque magnetometry which is further firmly supported by magnetic susceptibility, and EPR measurements. The study of the magnetization relaxation dynamics reveals field‐induced slow relaxation of magnetization due to the predominant Raman relaxation process. Theoretical calculations on **1**–**3** and optimized model complexes of **1** reveal insights into the electronic structure and highlight the impact of steric and electronic effects on modulating the *D* values. Overall, the studies reported pave the way for designing a new generation of Co^II^ complexes with enhanced  axiality and a lower rhombicity.

## Graphical abstract

We successfully quantify the zero field splitting parameter (*D*) in a series of five‐coordinate Co^II^ complexes, [Co(L)(X_2_)]∙CHCl_3_ (X = Cl (**1**), Br (**2**), I (**3**)) using torque magnetometry, while highlighting the role of halides in modulating *D* values. Additionally, ab initio calculations provide insights into the electronic structure and the steric and electronic effects influencing *D* in these complexes.

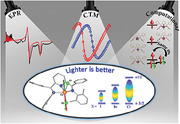

## 1 Introduction

Modulating and controlling a material's magnetic anisotropy is an active area of research for designing magnetic materials with improved properties.^[^ ^1^, ^2^ ^]^ This is particularly important for molecular‐based systems characterized by slow relaxation of the magnetization, called single molecule magnets (SMM).^[^ ^3^, ^4^, ^5^, ^6^ ^]^ Achieving an optimal design of SMMs is a very active area of research for various potential applications such as high‐density information storage devices, molecular spintronics or spin valves etc.^[^ ^7^, ^8^, ^9^, ^10^ ^]^ While in lanthanide complexes large MA is achieved by exploiting the deeply buried 4f‐orbitals,^[^ ^11^, ^12^ ^]^ quenching of orbital angular momentum by the ligand field is a known issue that hampers increasing the magnetic anisotropy  in 3d‐transition metal complexes. To increase the magnetic anisotropy in 3d‐transition metal, the reduction of the coordination number around the 3d‐metal ion proved to be a successful synthetic strategy.^[^ ^13^ ^]^ By exploiting this approach, record anisotropic barriers have been reported for two‐coordinate Fe^I^ (226 cm^−1^) and Co^II^ (450 cm^−1^) complexes.^[^ ^14^, ^15^ ^]^ While these complexes constitute interesting case‐studies and greatly enhanced our understanding of magnetization relaxation dynamics, their high reactivity and lack of stability under ambient conditions make them unsuitable for practical molecular‐based devices.

On the other hand, ambient stability in 3d metal ion complexes typically requires a minimum coordination number of four or more, though this often comes at the expense of reduced orbital angular momentum.^[^ ^16^ ^]^ However, extensive research has been conducted on modulating both the type and the magnitude of the MA in tetrahedral Co^II^ complexes, with various intricate factors significantly influencing these properties. Examples include the nature of donor atoms (hard or soft) around the metal ion, peripheral substituents on the ligand, cations or anions in the crystal lattice that modulate metal ion geometry via supramolecular interactions, and ligand systems that enforce D_2d_ symmetry around the Co^II^ ion regardless of donor atom types.^[^ ^17^, ^18^, ^19^, ^20^, ^21^, ^22^ ^]^

Penta coordination is also extremely interesting for designing high‐performance SMM. In a pioneering study on trigonal bipyramidal Co^II^ complexes Mallah and co‐workers demonstrated that strong σ‐donors at the axial position combined with soft donors at the equatorial sites enhance the easy‐axis character of magnetic anisotropy.^[^ ^23^ ^]^ Conversely, there are very few reports on the square pyramidal geometry of five‐coordinate Co^II^.^[^ ^24^, ^25^, ^26^ ^]^ Developing a structure‐property correlation for these ambient‐stable complexes is therefore an urgent issue and remains an active area of research. However, isolating a family of isostructural square pyramidal complexes is a major bottleneck in advancing magneto‐structural correlations.

Another significant challenge in this field is the quantitative determination of magnetic anisotropy  in mononuclear anisotropic complexes, such as Co^II^ ions. Since magnetic anisotropy is intrinsically linked to spatial orientation, angular‐resolved measurements on single crystals are often required to determine both its magnitude and orientation. Techniques such as single‐crystal magnetometry are powerful but are primarily effective for molecular systems containing only collinear contributions to the magnetic anisotropy and are often limited to low‐temperature studies due to the dominance of isotropic contributions over the anisotropic ones at high temperature.^[^ ^27^ ^]^ Other conventional techniques, such as electron paramagnetic resonance (EPR), are often limited by the presence of strict selection rules and the fact that the magnitude of the anisotropy typically exceeds the instrument's frequency range.^[^ ^28^ ^]^ Although sophisticated methods like polarized neutron diffraction (PND) are available for this scope, access to large scale facilities is limited, and large single crystals are required for neutron diffraction studies, posing additional challenges in this area of research.^[^ ^29^ ^]^

In contrast, cantilever torque magnetometry (CTM) offers unparalleled sensitivity to the magnetic anisotropy of metal complexes, allowing it to disentangle non‐collinearities within any crystal system and offering the possibility to study small single crystals up to room temperature.^[^ ^30^ ^]^ In this study, we employed CTM to quantitatively determine the magnetic anisotropy of a series of square pyramidal Co^II^ complexes with the general molecular formula [Co(L)(X)_2_]∙CHCl_3_, where L represents a Schiff base ligand ([2,6‐bis{1‐[(2,6‐diisopropylphenyl)‐ imino]benzyl}pyridine]) and X is Cl (**1**), Br (**2**), or I (**3**). Our detailed investigation reveals that all the complexes exhibit a large easy‐axis type magnetic anisotropy, which contrasts with most square pyramidal complexes reported in the literature.^[^ ^31^, ^32^, ^33^, ^34^ ^]^ and complex **1** has the highest *D* value among the largest reported for square pyramidal Co^II^ complexes.^[^ ^35^, ^36^ ^]^ Our systematic study shows that magnetic anisotropy decreases as the lighter halide is replaced with a heavier one (**1** > **2** > **3**). The EPR measurement performed on magnetically dilute solid solution of **2** further confirms the presence of an easy axis magnetic anisotropy. All three complexes exhibit field‐induced slow relaxation of the magnetization, and theoretical calculations performed on all complexes provide further insights into their electronic structures and elucidate the origin of the easy axis magnetic anisotropy in these systems.

## 2 Results and Discussion

**Figure 1:**
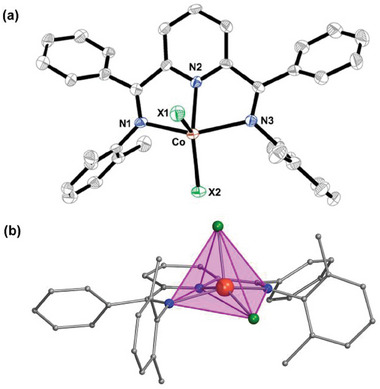
a) Thermal ellipsoid (50% probability) ORTEP representation of the molecular structure of **1**, representative for all the structures of **1**–**3** b) Polyhedral view showing the distorted square pyramidal geometry around Co^II^ ion. Orange = Co, Blue = N, Green = halide. For clarity, the hydrogen atoms, the solvent molecule, and the side chain carbon atoms are omitted.

The reaction of CoX_2_ (X = Cl or Br or I) with the NNN‐pincer ligand L in toluene followed by the crystallization of the complex in chloroform and hexane mixture yielded block‐shaped brown color crystals that are suitable for single‐crystal X‐ray diffraction (Scheme S1, Supporting Information). The single crystal data collection and structure solution reveals the molecular formula as [Co(L)(X_2_)]∙CHCl_3_ (where X = Cl (**1**), Br (**2**), or I (**3**)). All three complexes crystallize in the triclinic system. More specifically, **1** and **2** crystallize in the *P1* polar space group while **3** crystallizes in the *P‐1* non‐polar space group (Table S1, Supporting Information). Structural distortions disrupt complete symmetry, and the inclusion of a CHCl₃ solvent molecule within the crystal lattice induces chirality which, in turn, leads to the adoption of a polar space group. In **1** and **2**, the entire molecule corresponds to the asymmetric unit, whereas in **3**, two crystallographically distinct but structurally similar molecules are present in the asymmetric unit. Each complex is a Co^II^ monomer whose five coordination sites are occupied by one NNN pincer ligand and two halide ions (**Figure** **1**; Figure S1, Supporting Information).

The coordination geometry around the Co^II^ ion is closer to the distorted square pyramidal geometry than to the distorted trigonal bipyramidal geometry, which was confirmed by Continuous Shape analysis measurement (CShM) (Table S2, Supporting Information).^[^ ^37^ ^]^The three nitrogen donors of L and a halide ion around the Co^II^ ion are in near planar arrangement at the equatorial sites. In **1**–**3**, Co^II^ ion resides above the basal mean {N_3_X} plane by 0.57, 0.54, and 0.46 Å, respectively. The out‐of‐plane movement of the Co^II^ ion is dictated by the type of substituents on the imino (─C═N) carbon atom of the pincer ligand and the co‐ligands bound to the Co^II^ ion. In **1**–**3**, the Co‐N_pyridinic_ bond distance is found to be 2.036(2) Å, 2.038(3) Å, and 2.035(4) Å, respectively. On the other hand, the Co‐N_imino_ bond distance is ≈0.2 Å larger than the Co‐N_pyridinic_ bond distance, i.e., the average Co─N_imino_ bond length is observed to be 2.227 Å (for **1**), 2.217 Å (for **2**), 2.205 Å (**3a**) and 2.187 Å (**3b)**. In all three complexes, the Co─halide bond distances are larger than the Co─N bond distances. However, it is noticed that the Co‐X distance along the axial direction is ≈0.1 Å larger (Co─X_axial_ bond distance range 2.319–2.672 Å) than the Co‐X in the equatorial direction (Co‐X equatorial bond distance range 2.241–2.586 Å). Not surprisingly, Co‐X distance increases following the halide ionic radius. The selected bond distances and bond angles are provided in Table S3 (Supporting Information). In all the complexes, a chloroform molecule is involved in intermolecular H‐bonding besides the halide ions bound to the Co^II^ ions (Figure S2, Supporting Information). The complete list of atoms that are involved in intermolecular H‐bonding is detailed in Tables S4–S6 (Supporting Information). To check the bulk phase purity, we have measured powder X‐ray diffraction (PXRD) analysis, matching with the simulated pattern obtained from single crystal‐Xray measurements (Figure S3, Supporting Information).

Several square pyramidal Co^II^ complexes were reported in the literature, but a precise determination of the Zero Field Splitting (ZFS) parameters is very scarce for this geometry. Nevertheless, to understand the type of magnetic anisotropy associated with complexes **1**–**3**, we performed variable temperature X‐band continuous wave‐EPR (cw‐EPR) on the pure polycrystalline samples at 5.0 K (Figure S4, Supporting Information).

**1** and **2** give a broad EPR signal ranging from 100 to 400 mT (Figure S4, Supporting Information) while **3** did not provide any EPR signal. This behavior likely arises from the intrinsic fast relaxation associated with high spin Co^II^ ion and/or intermolecular dipolar coupling. For **3,** the presence of two crystallographically inequivalent molecules in the unit cell adds a further contribution to broadening, thus resulting in complete loss of EPR signal. To reduce the effect of the dipolar interaction, we have measured X‐band cw‐EPR of magnetically diluted solid solution of [Zn_0.9_Co_0.1_(L)Br_2_] (**2‐dil**) at 5.0 K**. 2‐dil** was obtained by using the corresponding diamagnetic analog [Zn(L)Br_2_], which is isostructural to **2** (Table S1, Supporting Information). The diamagnetic analog of **3** could not be obtained.

**Figure 2:**
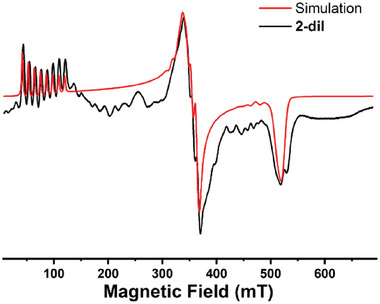
The X‐band EPR spectra of **2‐dil** recorded at 5.0 K (black trace). The red trace is the simulation of the experimental data using the parameters described in the main text. Experimental parameters: ν = 9.3964 GHz; Modulation amplitude = 0.3 mT; microwave power = 6.722 mW.

**2‐dil** shows a well‐resolved EPR spectrum (**Figure** **2**), which exhibits resonances in three distinct field regions, this can be interpreted as arising from an effective spin doublet in the presence of an anisotropic hyperfine interaction with the ^59^Co^II^ nucleus (*I* = 7/2; 100% abundance). The very low field at which the first resonance is observed compared to the other two indicates that the anisotropy of the ground doublet has an easy axis nature. The experimental EPR spectrum of **2‐dil** was satisfactorily simulated with the following spin Hamiltonian within the effective *S*’ = ½ formalism (Equation 1).^[^ ^38^ ^]^

(1)

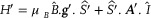




An excellent agreement between the experimental EPR spectrum and the best simulation was obtained using the following parameters $g_{z}^{\prime }=8.31\left(1\right),\;\;g_{y}^{\prime }=1.89\left(2\right),\;and\;\;g_{x}^{\prime }=1.30\left(2\right);$ and the hyperfine coupling tensor of $A_{z}^{\prime }=1300\left(5\right)\;MHz,\;\;A_{y}^{\prime }=100\left(5\right)\;MHz,\;and\;\;A_{x}^{\prime }=40\left(5\right)\;MHz$ and assuming collinearity of the two magnetic tensors. This pattern ($g_{z}^{\prime }\gg \;g_{y}^{\prime },\;g_{x}^{\prime }$) is consistent with those of other mononuclear Co^II^ complexes reported in the literature that exhibit axial anisotropy.^[^ ^39^, ^40^ ^]^

The standard X‐band EPR frequency (h*ν* ∼ 9 GHz ∼ 0.3 cm^−1^) is not sufficient to access inter doublet transitions of the S = 3/2, therefore to determine the magnetic anisotropy associated with all three complexes quantitatively, we have utilized CTM measurements. This technique is extremely powerful in delivering information about the orientation of the magnetic reference frame as well as about the ZFS parameters.

To perform CTM experiments an indexed single crystal was mounted on a piezoelectric cantilever torque magnetometer (see Supporting information for the experimental details). The sample has been measured in a wide range of temperatures (2–250K) and magnetic fields (2–9 T). During each measurement, the crystal has been rotated 180° around an axis perpendicular to the external magnetic field (Figures S5–S13, Supporting Information). The same procedure has been applied on single crystals of **1**, **2**, and **3**.

**Figure 3:**
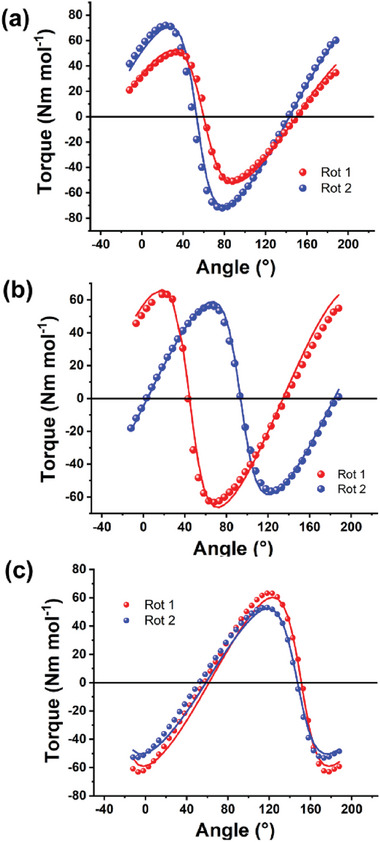
Torque signals for **1**, **2,** and **3** were obtained at 2 K and in a magnetic field of 4 T. The solid line are the best‐fit curves. The red and blue data points represent the first and second rotations, respectively.

In **Figure** **3** we report the torque curves obtained at *T* = 2 K and under applied magnetic field *B* = 4 T for all the studied crystals. All the curves show the typical shape expected for crystals containing only one inequivalent Co^II^ complex (i.e., zeros spaced of 90°, and mirror symmetry with respect to the x‐axis). While this is trivial for **1** and **2**, it also supports the notion that the two crystallographically inequivalent molecules in **3** are essentially identical and virtually related by a simple translation (in agreement with the structural discussion reported above).

**Figure 4:**
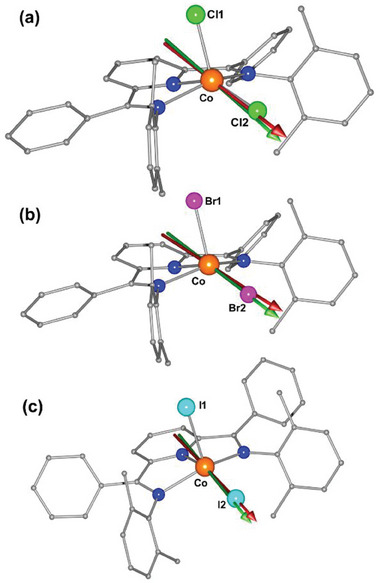
The g_z_ orientation was determined from CTM (green arrow) and from the computational calculations (red arrow) for complexes **1**–**3**.

To gain information about the magnetic reference frame of the samples we performed fits on the low‐temperature curves (2–5 K) using the Effective Spin Hamiltonian (Equation 1), without considering the hyperfine coupling, that is negligibly small with respect to the Zeeman term at the studied fields (Figures S5–S7, Supporting Information). For all the derivatives, the fitting parameters included the Euler angles describing the orientation of the magnetic reference frame,^[^ ^27^ ^]^ and the principal *g* values using (whenever possible) the values obtained by EPR as an initial guess. The obtained orientation of the easy‐axis is reported in **Figure** **4** while the complete reference frame is reported in Figure S14 (Supporting Information). It is interesting to notice that the orientation of the easy‐axis lies almost along the Co─X bond (with X being the halide belonging to the base of the square pyramidal coordination environment) in all three systems. That is peculiar considering the highly distorted coordination environment of the metal center.

Once we had obtained the position of the magnetic reference frame, we fitted all the data using the following Spin Hamiltonian operating on an *S* = 3/2:

(2) $${\hat{H}}_{s}=\mu_{B}\;\cdot \hat{S}\cdot g\cdot \vec{B}+D\left[{\hat{S}}_{z}^{2}-\frac{1}{3}S\left(S+1\right)\right]+E\left({\hat{S}}_{x}^{2}-{\hat{S}}_{y}^{2}\right)$$

Here *D* and *E* are the standard Zero‐Field splitting parameters representing the axial and rhombic component of the magnetic anisotropy tensor ***D***, and the ***D*** and ***g*** reference frames were assumed to be collinear.

The three main values of the ***g*** matrix of the Spin Hamiltonian of an easy‐axis *S* = 3/2 can be related to ***g*** ′ through the following equations (with η  =  *E*/*D*). ^[^ ^30^, ^41^, ^42^ ^]^

(3)

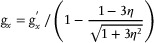



(4)

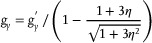



(5)

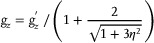




Therefore, the values *g_x_*, *g_y_*, and *g_z_* were obtained by fixing $g_{x}^{\prime },\;g_{y}^{\prime }$ and $g_{z}^{\prime }$ to those obtained previously (see **Table** **1**). Overall, our model well describes both the magnitude and shape of the torque curves obtained at all temperatures (Figures S5–S13, Supporting Information for all the data and fits) and the best fit Spin Hamiltonian (SH) parameters (g, *D*, *E/D* etc) are reported in Table 1.

**Table 1:** Spin Hamiltonian parameters extracted from CTM and CASSCF‐NEVPT2 calculations for complexes **1–3**.

Complex	g values			SH parameters		
	g_x_	g_y_	g_z_	D [cm^−1^]	❘E/D❘	Method
1	2.25 (3)	2.68 (3)	3.06 (2)	−72 (2)	0.27 (2)	CTM
	1.8	2.1	3.27	−110	0.10	NEVPT2
2	2.20 (2)	2.28 (2)	2.95 (1)	−67 (1)	0.26 (1)	CTM
	1.97	2.30	3.00	−80.6	0.19	NEVPT2
3	2.14 (2)	2.23 (2)	2.64 (2)	−25 (1)	0.27 (2)	CTM
	2.10	2.20	2.50	−30	0.22	NEVPT2

The model obtained for all three samples consists of a negative *D* (easy‐axis type anisotropy) that decreases (in absolute value) from **1** to **3**. All the samples exhibit a strong rhombicity with an E/D∼0.27.

**Figure 5:**
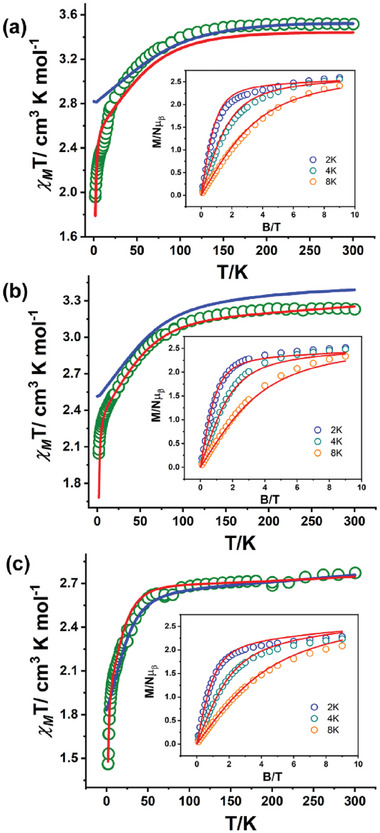
a–c) Temperature‐dependent magnetic susceptibility measurement was performed on the polycrystalline sample of complexes **1–3**, respectively in the presence of the 1 T external magnetic field. The solid blue line represents the simulation of the experimental magnetic data using the SH parameters derived from CASSCF/NEVPT2 calculations. Inset: Field‐dependent magnetization data collected on polycrystalline samples of complexes **1–3** at the indicated temperatures. The solid red lines in all the panels including the inset represent the best fit obtained using the parameters derived from CTM measurement described in the main text.

To further validate the quantitatively determined Spin Hamiltonian parameters of the model defined using CTM measurements, we have performed temperature‐dependent direct current (DC) magnetic susceptibility measurement of polycrystalline samples of **1**–**3** in the temperature range 2–300 K in the presence of a magnetic field of 1 T (**Figure** **5**).

The *χ_M_T* values of **1**–**3** at room temperature were found to be 3.51, 3.22, and 2.77 cm^3^ K mol^−1^ respectively. The experimentally observed *χ_M_T* value for all the complexes is significantly higher than the value for an *S* = 3/2 with an average *g* value of 2.0 (1.875 cm^3^ K mol^−1^). This is due to the significant residual orbital contribution to the overall magnetic moment of the complexes.^[^ ^35^, ^36^, ^43^, ^44^ ^]^

A similar trend is observed in the *χ_M_T* profile upon lowering the temperature for all the three samples, with a gradual decrease from room temperature down to 60 K. Below this temperature, *χ_M_T* sharply decreases to a final value of 1.95, 2.04, and 1.45 cm^3^ K mol^−1^ at 2.0 K for **1**–**3**, respectively. This decrease is attributed to the depopulation of the excited *m_S_* levels, i.e., to the magnetic anisotropy of the complexes.

Isothermal, field‐dependent magnetization measurements were performed at 2 K on polycrystalline samples of **1**–**3** up to 9 T (Figure 5‐inset). Upon turning on the magnetic field, the magnetic moment of all the complexes increases sharply. At 9 T the value of the magnetic moment reaches 2.59, 2.52, and 2.27 Nµ_B_ for **1**–**3,** respectively. Even at this field the absence of magnetic moment saturation in all the complexes indicates significant magnetic anisotropy, as confirmed by the non‐superimposable nature of the reduced magnetization plots (Figure S15, Supporting Information). Having extracted SH parameters from CTM measurements, we simulated the *χ _M_T*(*T*) data and *M*(*B*) data, achieving good agreement with the experimental results (Figure 5). For **2** and **3** we also introduced a small temperature‐independent paramagnetic factor (ca. 10^−4^ emu mol^−1^ for both systems) to account for the field‐induced mixing of excited states and correctly reproduce the high‐temperature slope of the *χ _M_T*.^[^ ^45^ ^]^ The estimated *D* value for complexes **1**–**3** ranks among the largest reported and is comparable to certain five‐coordinate Co(II) complexes (*D* = −77 cm⁻¹) described by Berry, Fiedler, and co‐workers.^[^ ^35^ ^]^ However, when compared to other geometries, such as certain four‐coordinate tetrahedral Co(II) complexes, even larger *D* values (*D* = −161 cm⁻¹) have been observed, as reported by Freedman and co‐workers.^[^ ^28^, ^46^ ^]^

**Figure 6:**
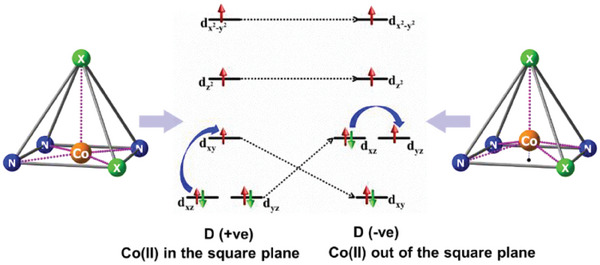
Simplified d‐orbital energy diagrams depicting square pyramidal coordination geometry with the metal ion positioned within the basal plane (left) and above the basal plane (right).

To understand the origin of the easy‐axis magnetic anisotropy (*D* < 0) in complexes **1–3**, we performed state‐average complete active space self‐consistent field (SA‐CASSCF) calculations based on the X‐ray crystal structure for complexes **1**–**3**. Our active space included 7 electrons in 5 orbitals CAS (7, 5), including all 10 quartet and 40 doublet contributions (see computational details in ESI). As stated in the structural description, all the complexes possess distorted square pyramidal geometry, and particularly, the Co^II^ ion sits above the basal mean plane. The effect of this structural distortion on the orbital ordering is qualitatively captured in **Figure** **6**.

The computed Spin‐Hamiltonian parameters, including *D*, ❘*E/D*❘, *g_x_, g_y_*, and *g_z_*, for complexes **1**–**3** are presented in Table 1. For all complexes, the *D* value is estimated to be negative: −110.9, −80.6, and −30.0 cm^−1^, while the ❘*E/D*❘ values are 0.105, 0.190, and 0.226, respectively. Although the computed *D* values follow the experimental trend (**1** > **2** > **3**), they appear slightly overestimated, possibly due to variations in metal‐ligand bonding, particularly Co‐halogen π bonding effects.^[^ ^47^ ^]^The cause for this deviation in this specific geometry has been rationalized elsewhere.^[^ ^24^, ^25^, ^48^ ^]^ The calculated g values indicate significant anisotropy. The orientation of the magnetic anisotropy axis direction (*D_ZZ_* and *g_z_* axis) is almost colinear, as assumed in the fit of CTM data, and these directions are along the Co‐X direction, which lies in the equatorial plane square pyramidal geometry of Co^II^ ion in all the complexes (Figure 4). The computed magnetic susceptibility and magnetization align well with the experimental observations, further supporting the reliability of the computed SH parameters (Figure 5; Figure S16, Supporting Information) (see Supporting Information for more computational section).

**Figure 7:**
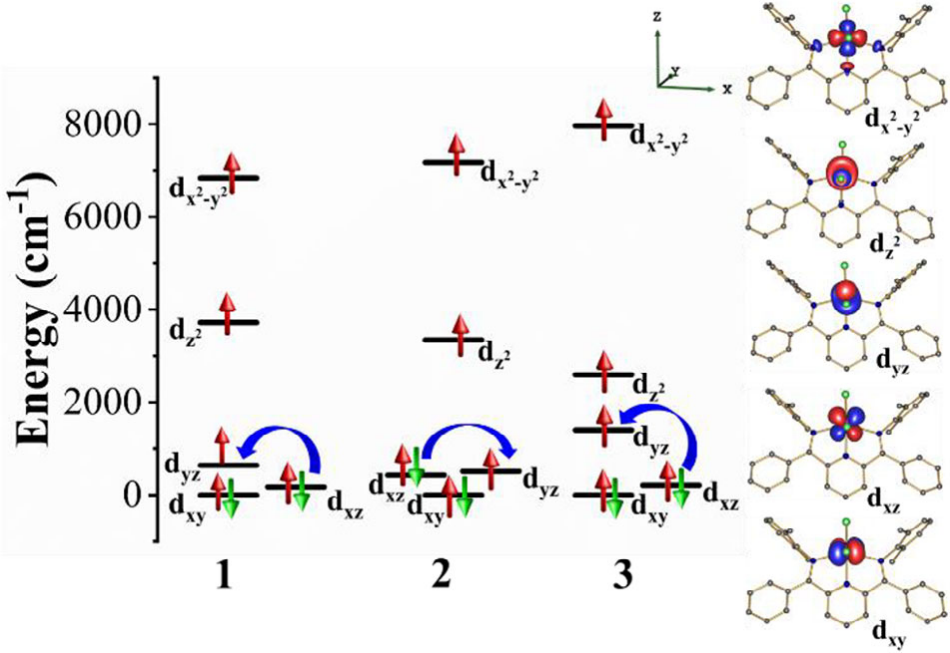
NEVPT2‐AILFT d‐orbital energy level diagram for complexes **1**–**3** with the d‐orbitals of complex **1** (right side) using iso‐surface value 0.0334.

**Figure 8:**
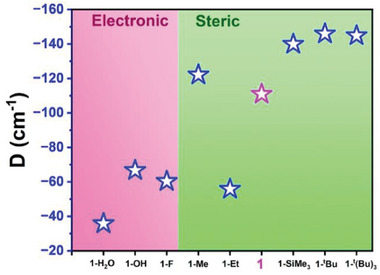
The computed *D* values of the model complexes derived from **1** and the value of **1** (magenta star) are also mapped for comparison purposes.

To rationalize the origin of the negative *D* values in all three complexes, we point out that the ground state wave function is highly multiconfigurational (Figure S17, Tables S7–S9, Supporting Information). This means that none of these states can be adequately described by a single determinant, indicating a pronounced mixing among the d‐orbitals due to the reduced symmetry.^[^ ^26^ ^]^ Consequently, the calculated d‐orbital splitting significantly deviates from what would be expected for an ideal TBP or SP geometries (**Figure** **7**). Near degeneracy of d_xy_, d_xz_, and d_yz_ orbitals lead to strong multi‐configurational character with the most dominating lowest energy transition/coupling being d_xz_ → d_yz_ (same ❘m*_l_* ❘ values), which contributes to negative D (Figure 7 and see the state‐wise contribution of *D* to the overall D value of the complex in Tables S7–S9, Supporting Information). In all three complexes, the lowest energy transition/coupling dominates over the second one, given the much higher energy of d_x_^2^_‐y_^2^, and thus determines the easy‐axis character of the magnetic anisotropy for all of them. Further, ab initio ligand field theory (AILFT, see Table S10 and also Figure S18, Supporting Information on bonding effects) reveals that nephelauxetic reduction in ζ is higher for complex **3**, indicating that it is more covalent than the other two complexes. This implies that large spin‐orbit coupling associated with heavier halides is not the sole factor increasing the ZFS of transition metal complexes; large metal‐ligand covalency drastically affects the nature of excitations, reducing the ZFS.^[^ ^49^ ^]^The systematic theoretical studies performed on complexes **1**–**3** reveal that the overall magnitude of the *D*‐value increases, as the out‐of‐plane shift of Co^II^ ion increases from the basal plane of square pyramidal geometry (**1** > **2** > **3**). To check the electronic and the steric influence of the system on the out‐of‐plane shift of the metal ion, we have performed calculations (CASSCF/NEVPT2) on optimized model complexes, which are derived from **1** (**Figure** **8**; Table S11, Supporting Information). To infer the electronic influence, we generated three models where the halide ions in **1** were replaced by a neutral water molecule (**1‐H_2_O**), fluoride (**1‐F**), and hydroxide (**1‐OH**). The remaining five models were built by systematically replacing the isopropyl group on the NNN‐ligand with a methyl group (**1‐Me**), ethyl group (**1‐Et**), tertiary butyl group

(**1‐^t^Bu**), tertiary butyl group at ortho, meta, and para position **(1‐^t^(Bu)_3_)** and trimethylsilyl group (**1‐SiMe_3_**) to study the steric influence. Here, we found that replacing the isopropyl substituent with the t‐butyl group enhances the *D*‐value significantly, and concurrently, rhombicity decreases with respect to the parent complex. This suggests a viable way to tune the geometry and therefore design molecular complexes with larger magnetic anisotropy.

**Figure 9:**
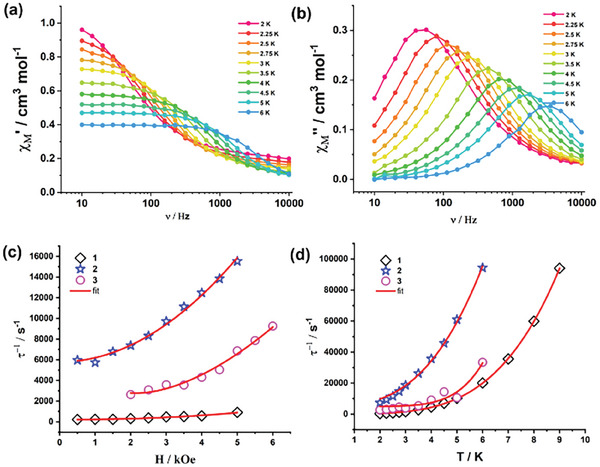
a,b) Frequency‐dependent in‐phase and out‐of‐phase susceptibility signals for complex **1** obtained at 2 K in presence of 0.2 T external DC bias c,d) Graphs *τ* ^−1^ versus H and *τ* ^−1^ versus *T* for **1**–**3**. Red lines are the fit of the data described in the main text.

Since all the complexes exhibit an easy axis of magnetic anisotropy, we probed their slow relaxation of the magnetization, using the alternating current (AC) magnetic susceptibility measurements on the polycrystalline samples in the presence of external dc field at 2 K and as a function of temperature in an external field of 0.2 T. The results for **1** are reported in **Figure** **9**a,b and those for **2–3** in Figures S19–S21 (Supporting Information). Through the fit of the experimental data using a generalized Debye model^[^ ^50^ ^]^ we obtained the relaxation time (*τ*) at different fields and temperatures for **1**–**3** (Tables S12–S14, Supporting Information). For **3,** we observe two peaks in the imaginary component of the ac susceptibility, probably due to the difference in the crystal structure compared to **1** and **2**. The second peak could be related to the presence of cooperative dipolar effect. However, one of them is outside the measurable range, so we have only analyzed the one where the peak was clearly visible. The field dependence and temperature dependence of τ is similar among the three samples (Figure 9c,d).

For all the three sample the mechanism is described by the sum of three different contributions,

(6) $$\;\tau^{-1}=\frac{b_{1}}{1+b_{2}H^{2}}+a_{1}H^{2}T+C\frac{e^{\frac{\hbar \omega }{kT}}}{{\left(e^{\frac{\hbar \omega }{kT}}-1\right)}^{2}}$$

The first contribution is Quantum Tunneling independent from temperature; then there is the Direct contribution and the Raman contribution. The best fit parameters are reported in **Table** **2**. The same set of parameters excellently reproduces both temperature and field dependent relaxation time plots (Figure 9c,d). It is interesting to notice that the Raman phonon frequency depends strongly upon replacing the lighter halide with a heavier halide.

**Table 2:** Raman phonon frequency obtained from the fit of magnetic relaxation mechanism.

Parameters	1	2	3
ℏ ***ω*** [***cm*** ^−1^]	78(2)	50(1)	109(2)
***a***_1_ [***Hz*** ***Oe*** ^−2^ ***K*** ^−1^)]	1.6(3) · 10^−5^	2.02(1) · 10^−4^	1.2(2) · 10^−4^
***b***_1_[***Hz***)	217(3)	5800(2)	4963(5)
***b***_2_ (***Oe*** ^−2^]	5.9(2) · 10^−8^	1.39(2) · 10^−13^	4.(3)4 · 10^−7^
**C** [***Hz***]	3.46(5) · 10^5^	4.1(1) · 10^5^	1.7(2) · 10^6^

This suggests that the molecular distortion promoting relaxation involves a Co‐X vibration, and its energy is expected to decrease increasing the atomic weight of the halogen atom. This trend is indeed observed between **1** and **2**, but not for **3**. Since the crystal structure of **3** is different compared to **1** and **2**, this discrepancy suggests that also the crystal lattice plays a significant role in tuning this frequency.

To infer the role of dipolar interactions, AC susceptibility measurements were performed on **2‐dil**, but it did not show a significant improvement on the relaxation time compared to its pure analog (Figure S22, Supporting Information for *H*_dc_  ≠ 0).

## 3 Conclusion

We characterized a series of isostructural square‐pyramidal Co^II^ complexes, [Co(L)(X_2_)]∙CHCl_3_ (X = Cl (**1**), Br (**2**), I (**3**)), using single‐crystal X‐ray diffraction. CTM measurements on oriented crystals revealed a decreasing trend in *D* values with increasing halide size: **1** (−72 cm⁻¹) > **2** (−67 cm⁻¹) > **3** (−25 cm⁻¹). Notably, complex **1** exhibits the largest axial anisotropy among known five‐coordinate Co^II^ square‐pyramidal complexes, contrasting with the predominantly easy‐plane anisotropy of most Co^II^ complexes in this geometry. The extracted SH parameters from CTM accurately reproduce the experimental magnetic data (*χ_M_T*(*T*) and *M*(*B*)), demonstrating the robustness of the measurements. The study also shows that the easy‐axis of all the complexes aligns with the Co–X direction, where X is in the equatorial plane, contrary to the expected alignment along the pseudo C_4_ axis of the square‐pyramidal geometry. Ab initio CASSCF/NEVPT2 calculations provide insight into the electronic structures of **1**–**3** and explain the observed *D* trends. Our magneto‐structural analysis identifies the Co^II^ ion's out‐of‐plane shift from the basal plane as the key geometric factor for achieving high negative *D* values, influenced by the bulky NNN pincer ligand. A recent high‐throughput computation study reveals that penta‐coordination has the potential to reach the largest |*D*| value ever reported for a Cobalt complex.^[^ ^16^ ^]^ Specifically, the most promising complex evaluated in the theoretical study exhibits a similar coordination sphere (2 halogen atoms and 3 nitrogen atoms), suggesting that chemical design (*i.e*., increasing the X‐Co‐X angle toward 180°) could lead to a massive increase of the magnetic anisotropy.^[^ ^16^ ^]^

## Conflict of Interest

The authors declare no conflict of interest.

## Supporting information



Supporting Information

## Data Availability

The data that support the findings of this study are available from the corresponding author upon reasonable request.
