# Supplementary material for: Quantifying Magnetic Anisotropy of Series of Five‐Coordinate CoII Ions: Experimental and Theoretical Insights
Source: Adv Sci (Weinh). 2025 Jan 14;12(9):2415624. doi: 10.1002/advs.202415624 (PMC11884563; doi:10.1002/advs.202415624)
Supplement: Supplementary file 1 — Supporting Information [file ADVS-12-2415624-s001.pdf]

ADVANCED SCIENCE
Open Access

Supporting Information

for *Adv. Sci.*, DOI 10.1002/advs.202415624

Quantifying Magnetic Anisotropy of Series of Five-Coordinate Co^{II} Ions: Experimental and Theoretical Insights

Vijaya Thangaraj, Daniele Sartini, Dipanti Borah, Deepanshu Chauhan, Vasudha Sharma, Lorenzo Sorace, Gopalan Rajaraman, Mauro Perfetti* and Maheswaran Shanmugam**

Quantifying Magnetic Anisotropy of Series of five-coordinate Co(II) ions: Experimental and Theoretical Insights

Vijaya Thangaraj,[#a] Daniele Sartini,[#b] Dipanti Borah,[a] Deepanshu Chauhan,[a] Vasudha Sharma,[a] Lorenzo Sorace,[b] Gopalan Rajaraman,*[a] Mauro Perfetti,*[b] Maheswaran Shanmugam*[a]

[a] Ms. V. Thangaraj, Ms. D. Borah, Mr. D. Chauhan, Ms. V. Sharma, Prof. Dr. G. Rajaraman, Prof. Dr. M. Shanmugam.
Department of Chemistry,
Indian Institute of Technology Bombay
Powai, Mumbai 400076, Maharashtra (India)
E-mail: rajaraman@chem.iitb.ac.in (GR); eswar@chem.iitb.ac.in (MS)

[b] Mr. D. Sartini, Prof. Dr. L. Sorace, Prof. Dr. M. Perfetti.
Dipartimento di Chimica “Ugo Schiff” and UdR INSTM
Università degli Studi di Firenze, Via della Lastruccia 3−13, 50019 Sesto Fiorentino, Italy
E-mail: mauro.perfetti@unifi.it

Materials and Methods

All of the reactions were carried out under an inert Argon atmosphere. Chemicals and solvents were purchased from commercially available sources (Sigma Aldrich/Thermofisher Scientific). They were used without further purification, except for aniline, which was freshly distilled before being used for ligand synthesis. Toluene was dried using the literature methods. The [2,6-bis{1-[(2,6-diisopropylphenyl)- imino]benzyl}pyridine]) ligand (NNN) was synthesized as per the literature report.[1]

Single crystal X-ray diffraction measurements:

Single crystal X-ray diffractions were performed on Bruker diffractometer with MoKα radiation (λ = 0.71073 Å). Cell refinement and data reduction were performed using CrysAlisPro 1.171.38.43. The Crystal data was solved by direct methods and then refined by full-matrix least-squares refinements based on F^2 using the SHELXL, as implemented in Olex2-1.5. All non-hydrogen atoms were refined anisotropically, and all hydrogen atom's positions were generated geometrically. Symmetry analysis was also performed using PLATON's ADDSYM tool. The data collection and structure refinement of these crystals are summarized in Table **S1** (CCDC number for **1**-**3**, respectively 2384288-2384290).

Powder X-ray diffraction measurement (PXRD):

Powder X-ray diffraction (PXRD) was performed on a RigakuD/tex Ultra 250 instrument using a Cu Kβ filter. The diffraction patterns were collected in the 2θ range of 5°−50° with a step size of 0.01.

Magnetic measurement:

Magnetic susceptibility measurements were carried out on a Quantum Design SQUID magnetometer MPMS-3 and PPMS, operated between 1.8 to 300 K for DC-applied fields ranging from 0 to 70 kOe and 0 to 90 kOe, respectively. Alternating current (AC) susceptibilities were carried out under an oscillating AC field with frequencies ranging from 0.1 Hz to 1 kHz (MPMS-3) and 10 Hz to 10 kHz (PPMS).

Cantilever Torque Measurement (CTM):

The sample has been measured in a wide range of temperatures (2-250 K) and magnetic fields (2-9 T). During each measurement, the crystal has been rotated 180° around an axis perpendicular to the external magnetic field. Considering the ab'c* reference frame commonly used in cantilever torque magnetometry,[2] the coordinates of the vector that indicates the direction of the magnetic field for the three samples at zero angle is [0.831; -0.518; -0.199], [-0.714; 0.452; 0.534] , [0; 0; 1] respectively for **1-3**. The rotation axis are indicates by the vectors [-0.125; 0.175; -0.977] (1° Rot) and [0.541; 0.837; 0.081] (2° Rot) for **1**, [0.451; 0.285; 0.844] (1° Rot) and [0.533; -0.844; 0] (2° Rot) for **2**, [-0.970; 0.244; 0] (1° Rot) and [0.244; -0.970; 0] (2° Rot) for **3**. The same procedure has been applied on single crystals of all the studied samples.

Computational Details

We conducted ab initio single-point calculations employing the ORCA 4.0.1 suite of programs. To understand the electronic and magnetic properties of the Co(II) ion, to extract zero-field splitting parameters, g-values, and energy states. The coordinates utilized were directly taken from the crystal structure without any geometry optimization. The Douglas-Kroll-Hess (DKH) Hamiltonian was applied to address scalar relativistic effects. In our calculations, we utilized DKH-contacted versions of basis sets: DKH-def2-TZVP for Co, DKH-def2-TZVP(-f) for N, and DKH-def2-SVP for the remaining atoms. During the orbital optimization phase, the state average complete active space self-consistent field (SA-CASSCF) method was employed, utilizing a CAS(7,5) active space comprising 7 electrons in 5 orbitals. All 10 quartet and 40 doublet states were computed within this active space. For dynamic electron correlation, the converged SA-CASSCF wavefunction was the basis for strongly contracted N-electron valence perturbation theory second-order (NEVPT2) calculations. Additionally, an ab initio ligand field theory (AILFT) analysis was conducted to obtain highly accurate d-orbital energies for the complex. To understand the direction of the magnetic anisotropy axis, we computed the LoProp charges on complexes **1**-**3** using CASSF/RASSI-SO calculations performed with the Molcas 8.2 suite of programs. We employed the TZVP basis set for Co (Co.ANO-RCC...6s5p3d2f1g), VTZ for Cl (Cl.ANO-RCC...5s4p2d), VTZ for C (C.ANO-RCC...4s3p2d), VTZ for N (N.ANO-RCC...4s3p2d), and VDZ for H (H.ANO-RCC...2s) in complex **1**. For complex **2**, the basis sets remained the same except that we replaced the Cl basis set with the TZVP basis set for Br (Br.ANO-RCC...5s4p1d). For complex **3**, the basis sets were the same as in complex **1**,

except that we replaced the Cl basis set with the VDZP basis set for I (I.ANO-RCC...6s5p3d1f). We chose seven electrons in five d orbitals, i.e., CAS (7,5), including 10 quartet and 40 doublet states in the RASSI-SO calculations. Furthermore, we used Gaussian16 to optimize the model complex by employing the unrestricted B3LYP functional and the LanL2DZ basis set for the Co ion, with a 6-31G* basis set for the rest of the atoms. This approach appears to be the most promising methodology for Co(II) complexes. [3]

Computational Section

The following equation can describe the sign and magnitude of the D values of all three complexes:

$$D_{ij} = -\frac{\zeta^2}{4S^2}\sum_{p,q}\frac{(\Psi_p|\hat{l}_i|\Psi_q)(\Psi_q|\hat{l}_j|\Psi_p)}{\varepsilon_q-\varepsilon_p} \qquad \textbf{(1)}$$

where, D_{ij} represents the diagonalized and traceless matrix components obtained from the second-order perturbation of spin-orbit coupling (SOC), ζ denotes the effective SOC constant of Co^{II} in the molecular environment, and Ψ_p and Ψ_q are the ground and excited state wavefunctions corresponding to the same spin value S, with ε_q and ε_p being their respective energies. [4] The sign of the D parameter in transition metal complexes is determined by the $|m_l|$ values of the orbitals involved in the coupling. When the spin-conserved transition involved between the orbital of the same $|m_l|$ values, the summation term given in equation **1** remains positive, and hence the resultant D value is negative. Conversely, if the transition between the orbitals with different $|m_l|$ values results in a positive D value.[5] Therefore, the lowest energy transition (between the orbitals) in a complex predominantly determines the sign of the D-value.

General Synthetic procedure to isolate 1-3:

CoX_2 (X = Cl or Br or I) (1 mmol) was added to a clear yellow solution of NNN-pincer ligand(L) (1 mmol) in 8 mL toluene. The reaction mixture was stirred overnight for **1** and refluxed for **2** and **3**. The residue obtained was then filtered, washed with hexane, and dried. Block-shaped single crystals suitable for X-ray analysis were obtained the next day by dissolving the residue in chloroform and layering it with hexane. (Yield of **1**-35%, **2**-24%, **3**-15%); Elemental analysis details: **1** Calc. (%) C, 61.80; H, 5.65; N, 4.91. Found (%) C, 61.62;

H, 5.53; N, 4.71. **2** Calc. (%) C, 55.98; H, 5.12; N, 4.45. Found (%) C, 55.82; H, 4.98; N, 4.27. **3** Calc. (%) C, 50.91; H, 4.66; N, 4.05. Found (%) C, 50.75; H, 4.55; N, 3.96.

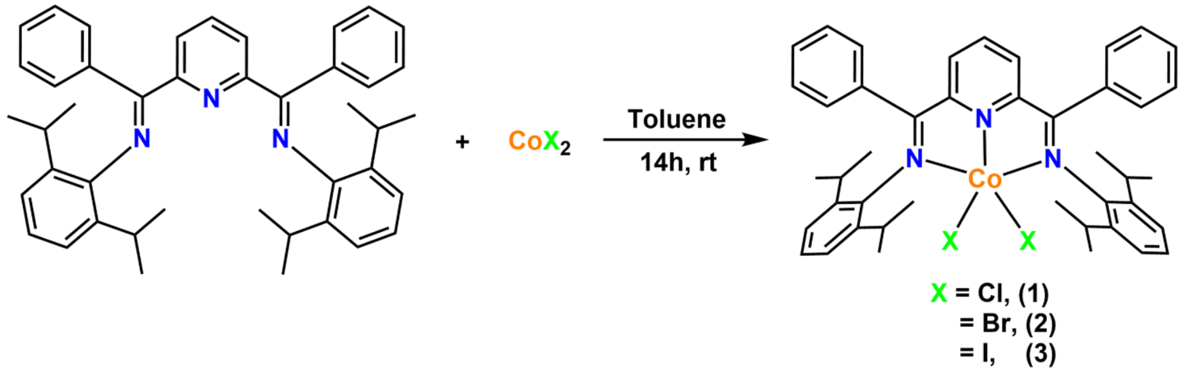

Scheme S1: Synthetic scheme followed to isolate **1-3**.

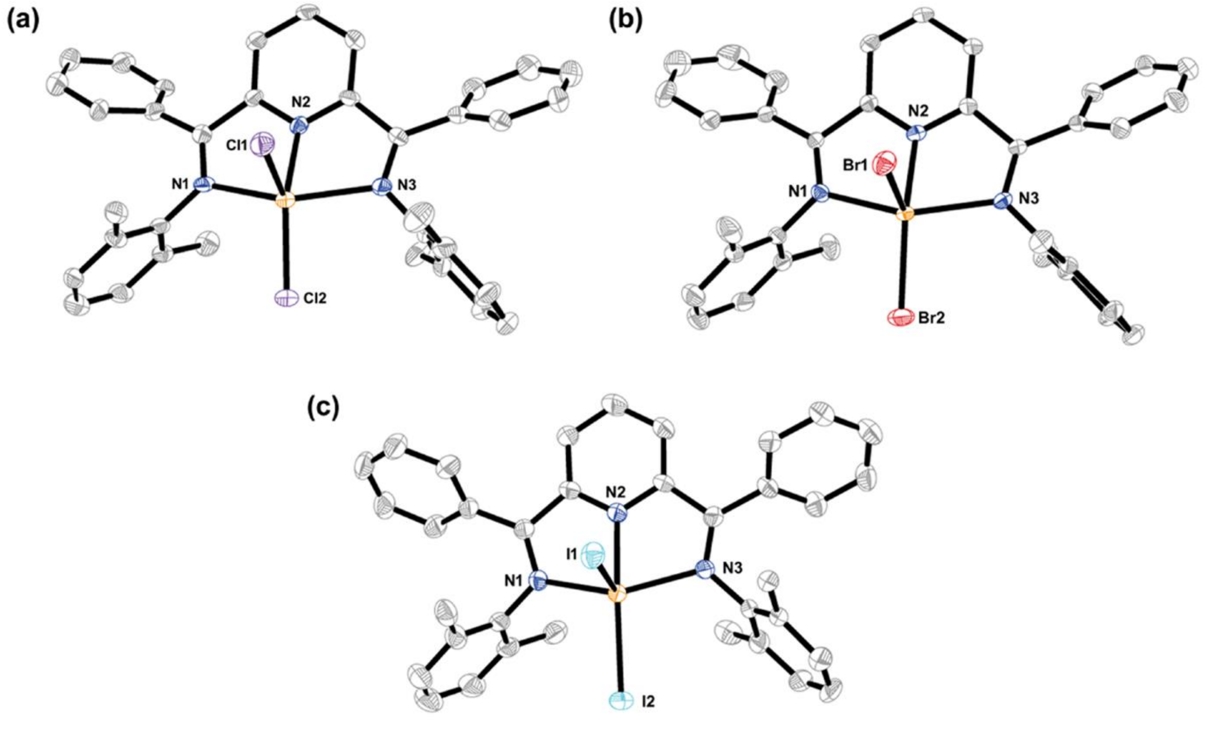

Figure S1: (a-c) Thermal ellipsoid (50% probability) ORTEP figure of **1-3**. The hydrogen atoms, methyl moieties, and solvent molecules are removed for clarity.

Table S1: Crystallographic parameters of **1-5.**

Complex	**1**	**2**	**3**	**4**
Empirical formula	$C_{44}H_{48}Cl_5CoN_3$	$C_{44}H_{48}Br_2Cl_3CoN_3$	$C_{87.75}Cl_{5.25}Co_2H_{95.75}I_4N_6$	$C_{44}H_{48}Br_2Cl_3N_3Zn$
Formula weight	855.03	943.95	1956.49	950.39
Temperature/K	123	150.15	150.15	150(2)
Crystal system	triclinic	triclinic	triclinic	triclinic
Space group	P1	P1	P-1	P1
a/Å	9.6097(3)	9.5892(2)	11.12730(10)	9.6329(3)
b/Å	9.6241(2)	9.7939(3)	18.2027(3)	9.7669(2)
c/Å	13.6732(2)	13.7013(4)	23.7064(3)	13.7150(4)
α/°	93.129(2)	93.829(2)	78.9470(10)	94.221(2)
β/°	107.610(2)	107.272(2)	80.1590(10)	107.175(2)
γ/°	114.313(3)	113.936(2)	75.9440(10)	114.153(2)
Volume/Å^3	1074.79(5)	1096.92(6)	4532.33(11)	1096.56(6)
Z	1	1	2	1
ρ_{calc}g/cm^3	1.321	1.4289	1.434	1.439
μ/mm^{-1}	0.744	2.428	1.860	2.598
F(000)	445.0	481.4	1952.0	484.0
Crystal size/mm^3	0.25 × 0.2 × 0.12	0.34 × 0.2 × 0.21	0.45 × 0.28 × 0.3	0.27 × 0.24 × 0.2
Radiation	Mo Kα (λ = 0.71073)	Mo Kα (λ = 0.71073)	MoKα (λ = 0.71073)	MoKα (λ = 0.71073)
2Θ range for data collection/°	4.74 to 49.98	4.66 to 50	3.142 to 49.998	3.188 to 49.996
Reflections collected	43021	59956	185786	36945
Independent reflections	7591 [R_{int} = 0.0498, R_{sigma} = 0.0299]	7753 [R_{int} = 0.1021, R_{sigma} = 0.0936]	15947 [R_{int} = 0.1456, R_{sigma} = 0.0510]	7723 [Rint = 0.0571, Rsigma = 0.0464]
Goodness-of-fit on F^2	1.042	1.035	1.045	1.015
R_1	0.0301	0.0412	0.0430	0.0375
wR_2	0.0752	0.0996	0.0863	0.0802

Table S2: Summary of SHAPE analysis for **1-3.**

Complexes	PP-5	vOC-5	TBPY-5	SPY-5	JTBPY
1	32.920	4.175	5.607	**2.225**	8.514
2	32.687	4.669	5.988	**2.611**	9.504
3	35.579	5.299	6.360	**3.329**	8.887

Table S3: Selected bond lengths and bond angles of **1-3.**

**Bond angle (°)**	**1**	**2**	**3**
X1-Co1-X2	122.80(5)	122.109(18)	109.48(2)
N1-Co1-X2	98.80(11)	97.32(8)	100.06(11)
N1-Co1-X1	99.38(11)	96.76(8)	101.60(10)
N2-Co1-X2	90.47(12)	148.50(8)	163.02(12)
N2-Co1-X1	146.67(12)	89.32(8)	87.44(12)
N3-Co1-X2	98.03(11)	99.62(8)	102.93(10)
N3-Co1-X1	96.83(12)	98.73(8)	101.09(10)
**Bond length(Å)**	**1**	**2**	**3**
Co1- X2	2.241(13)	2.367(5)	2.583(7)
Co1- X1	2.319(13)	2.465(4)	2.671(7)
Co1-N1	2.235(4)	2.213(3)	2.2124)
Co1-N2	2.036(4)	2.038(3)	2.037(4)
Co1-N3	2.219(4)	2.222(3)	2.198(4)

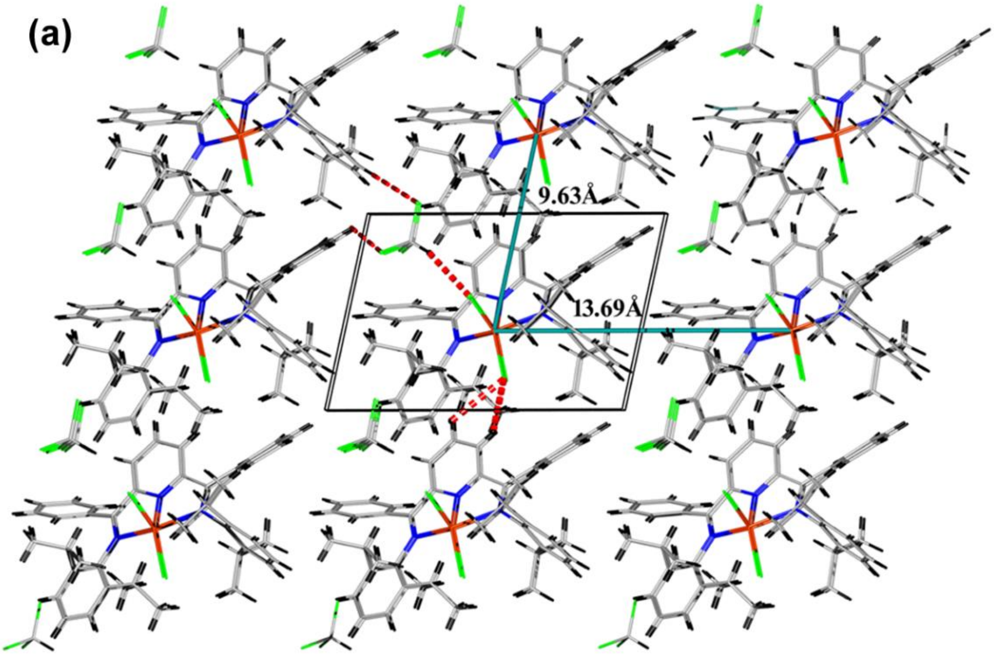

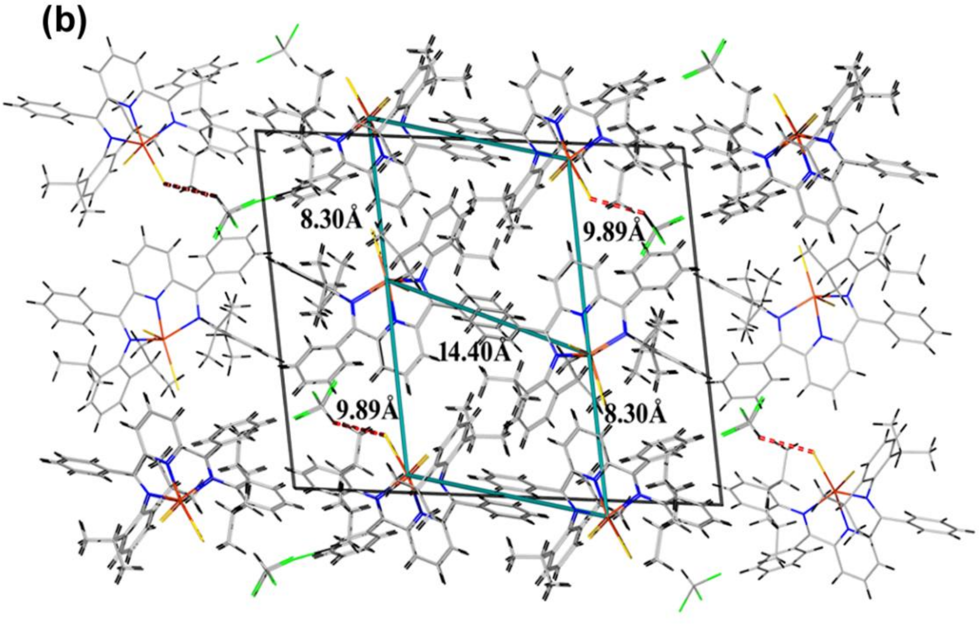

Figure S2: Packing diagrams of **1** (panel a) and **3** (panel b). The dotted red lines represent the intermolecular hydrogen bonding. Turquoise green solid lines represent the intermolecular distance between Co(II) ions. Color code: brown, Co(II); green, Cl; yellow, I; grey, C; blue, N; Black, H.

Table S4. Atoms involved in intermolecular hydrogen bonding and its corresponding bond distance and bond angles in **1.**

**H-Bond Donor(D)-Acceptor(A)**	**D...A (Å)**	**∠(DHA) (°)**
C33-H33...Cl2_$1	3.718(9)	138.8
C44-H44...Cl2_$1	3.646(11)	163.6
C34-H34C...Cl1_$1	3.869(10)	166.9
C38-H38...Cl2_$2	3.767(9)	152.6

Symmetry operators for generating equivalent atoms: $1 = +X, +Y, +Z, $2 = 1+X, 1+Y, +Z.

Table S5. Atoms involved in intermolecular hydrogen bonding and its corresponding bond distance and bond angles in **2.**

**H-Bond Donor(D)-Acceptor(A)**	**D...A (Å)**	**∠(DHA) (°)**
C10-H10...Br1_$1	3.800(7)	137.3
C44-H44...Br1_$1	3.721(10)	167.7
C12-H12B...Br2_$1	3.999(8)	162.1
C43-H43A...Br2_$1	3.857(9)	146.6
C18-H18...Br1_$2	3.777(7)	136.3
C5-H5...Br1_$3	3.800(7)	150.8
C23-H23...Br2_$4	3.507(6)	128.5

Symmetry operators for generating equivalent atoms: $1 = +X, +Y, +Z, $2 = 1+X, +Y, +Z, $3 = 1+X, 1+Y, +Z, $4 = +X, Y-1, +Z

Table S6. Atoms involved in intermolecular hydrogen bonding and its corresponding bond distance and bond angles in **3.**

**H-Bond Donor(D)-Acceptor(A)**	**D...A (Å)**	**∠ (DHA) (°)**
C34-H34A...I1_$1	3.778(5)	118.1
C10-H10...I1_$1	4.017(6)	137.3
C53-H53...I3_$1	3.993(5)	134.6

Symmetry operators for generating equivalent atoms: $1 = +X, +Y, +Z

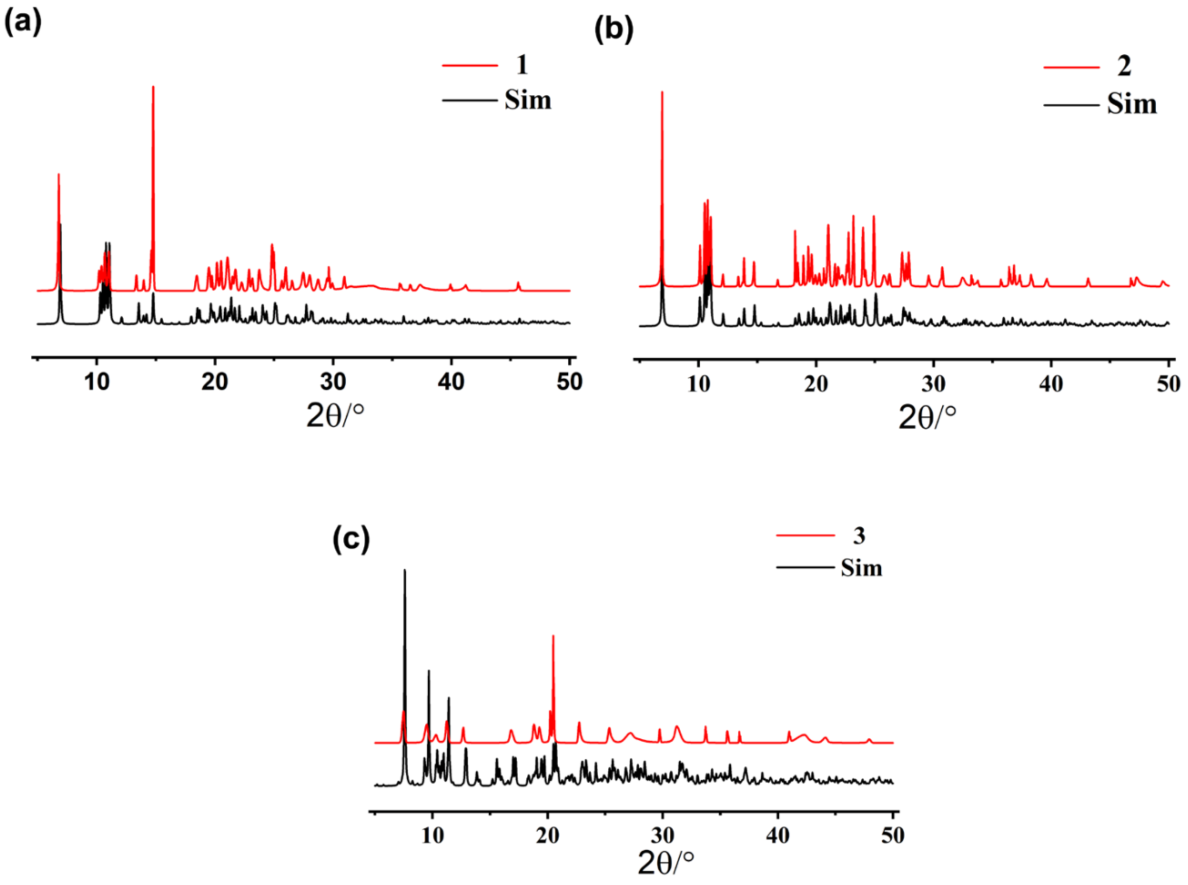

Figure S3: PXRD patterns of **1**,**2** and **3** (a-c).

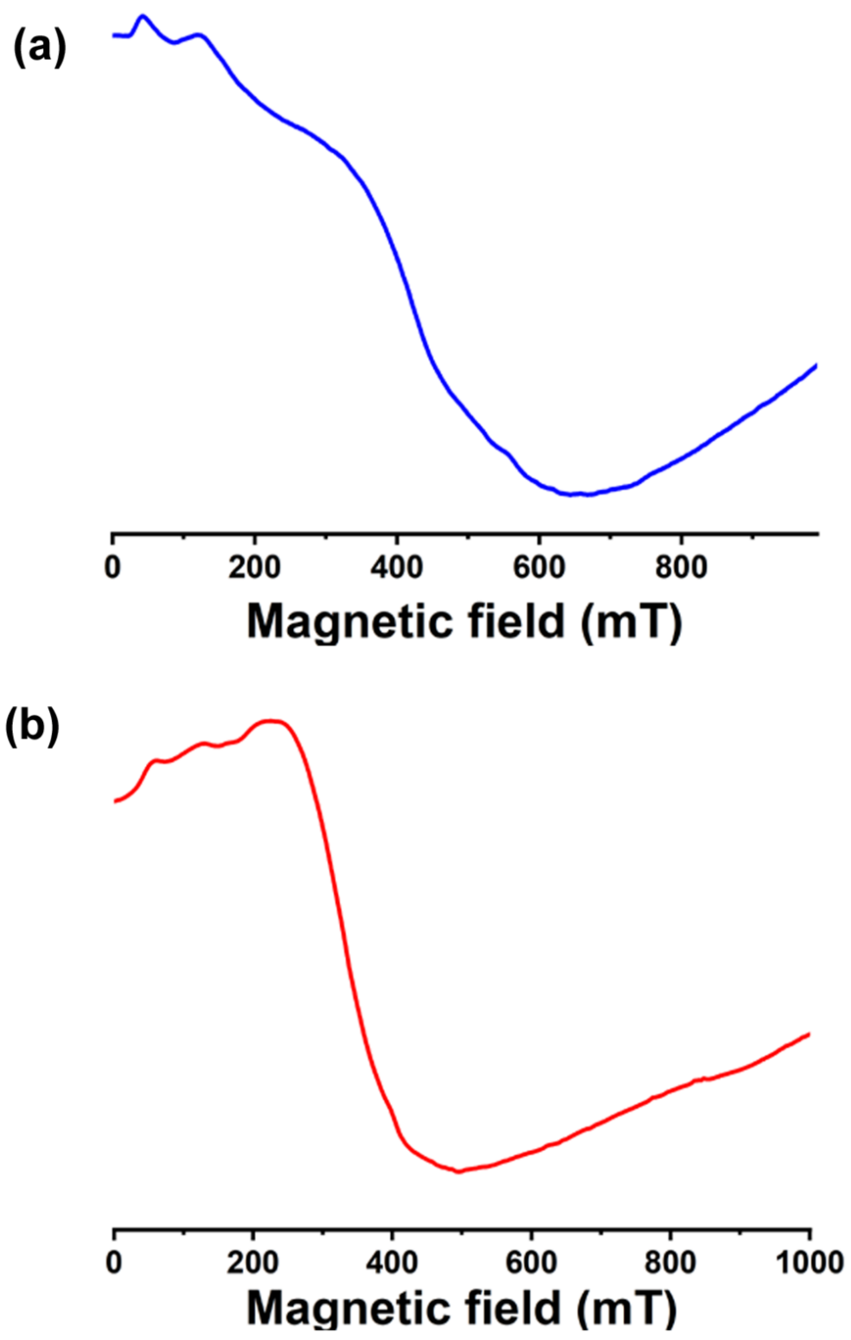

Figure S4: X-Band EPR spectrum of 100% polycrystalline samples of **1** and **2** at 5 K.

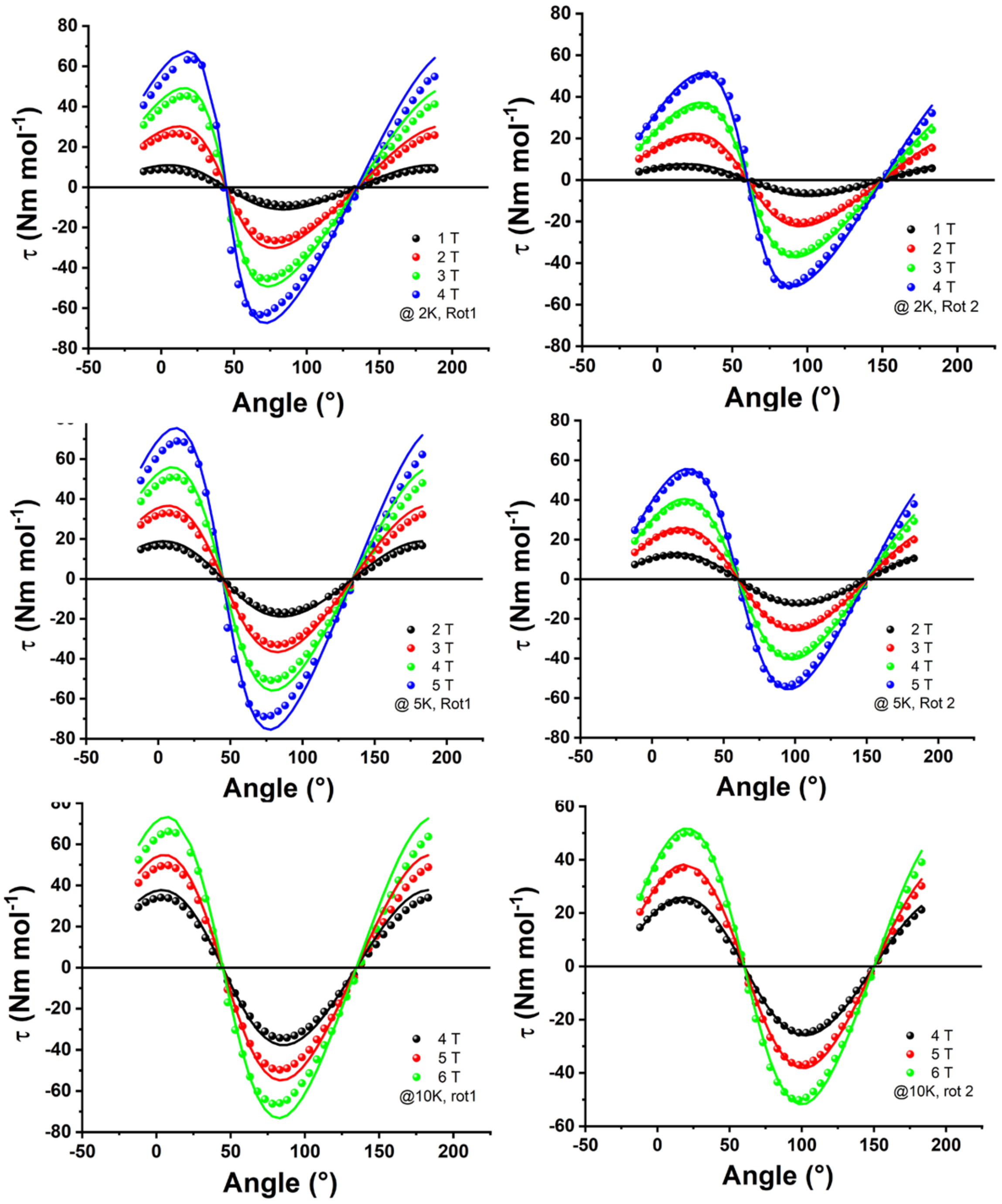

Figure S5: Torque curves obtained at 2-10 K temperatures and at indicated fields for **1**.

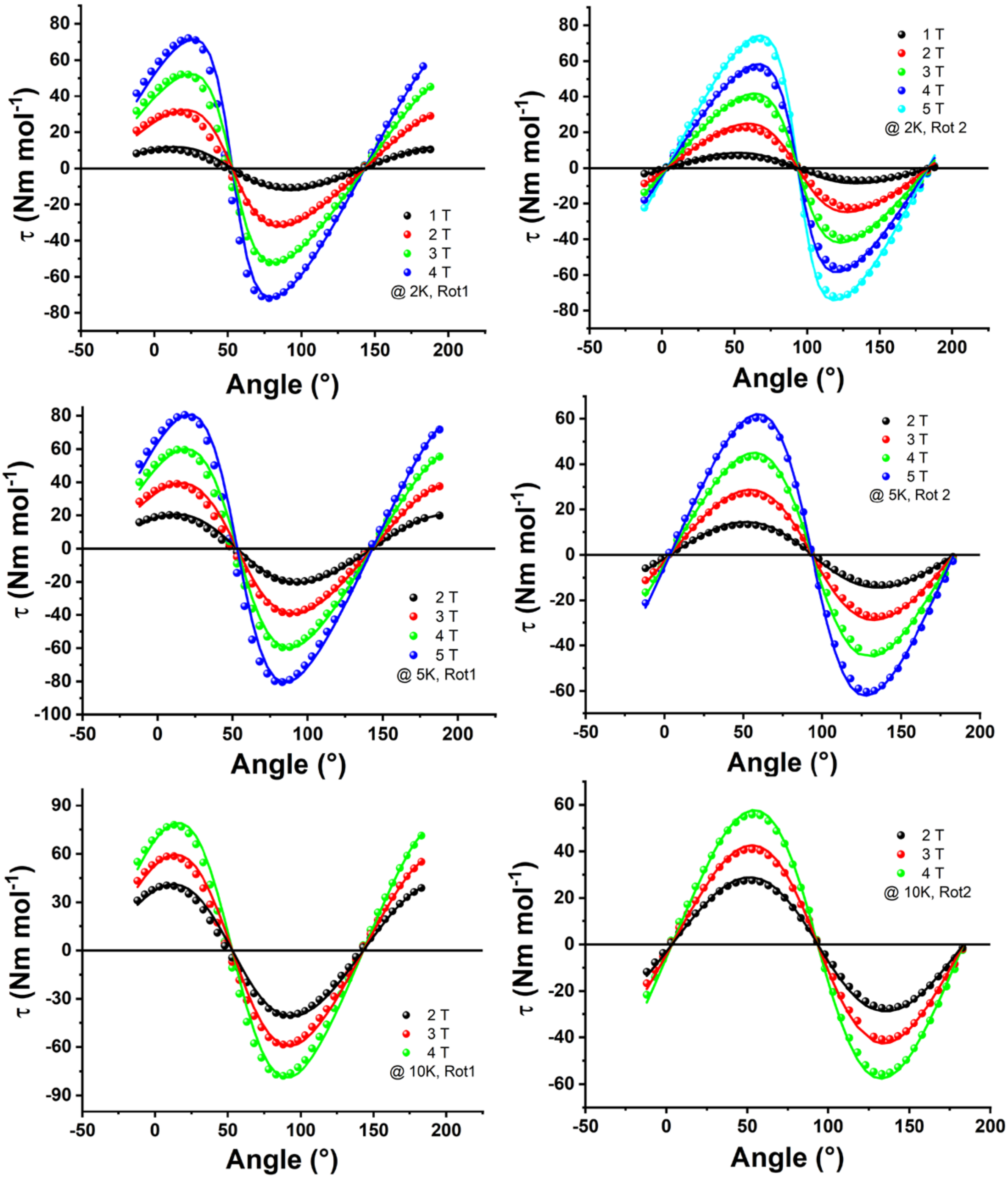

Figure S6: Torque curves obtained at 2-10 K temperatures and at indicated fields for **2**.

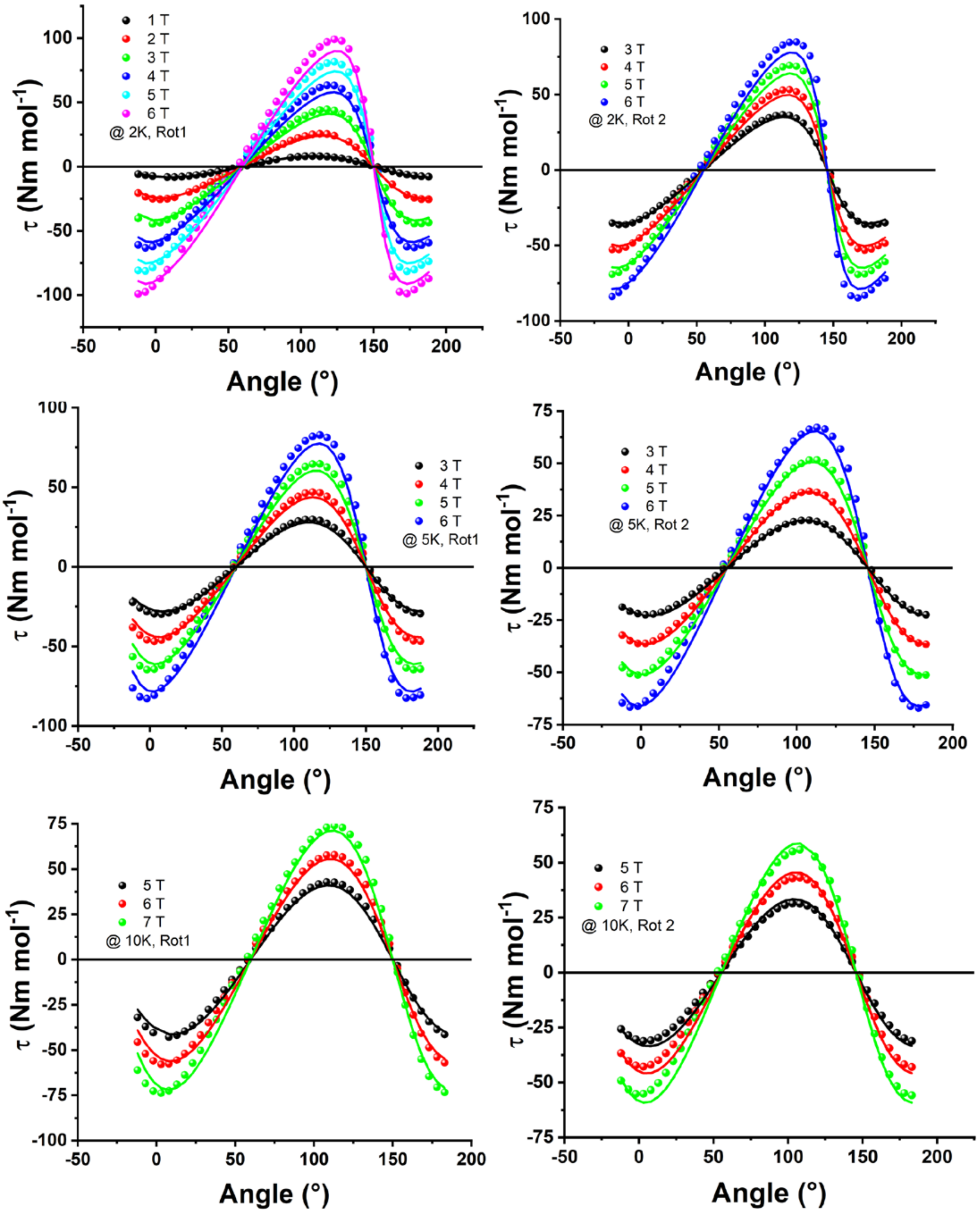

Figure S7: Torque curves obtained at 2-10 K temperatures and at indicated fields for **3**.

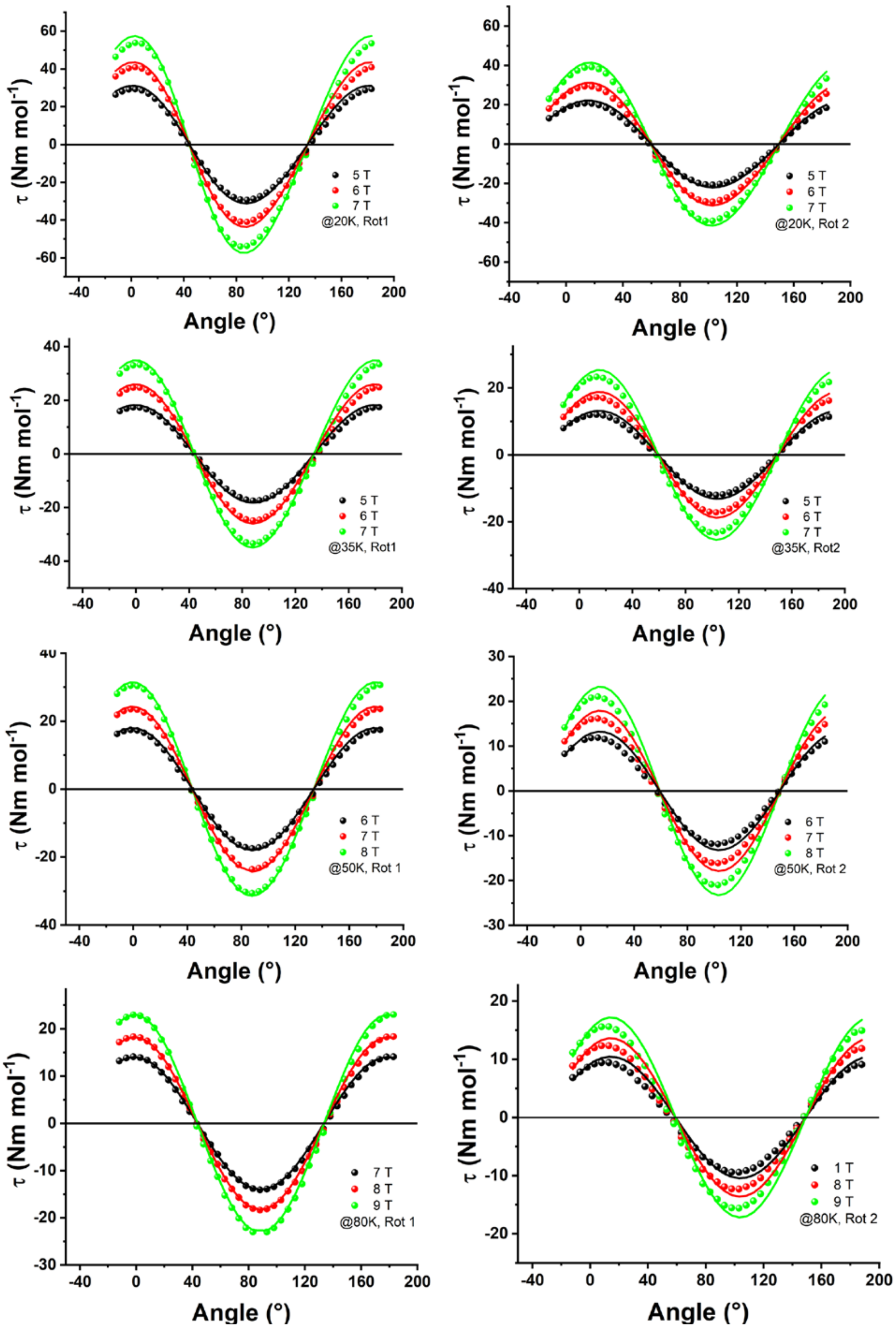

Figure S8: Torque curves obtained at 20-80 K temperatures and at indicated fields for **1**.

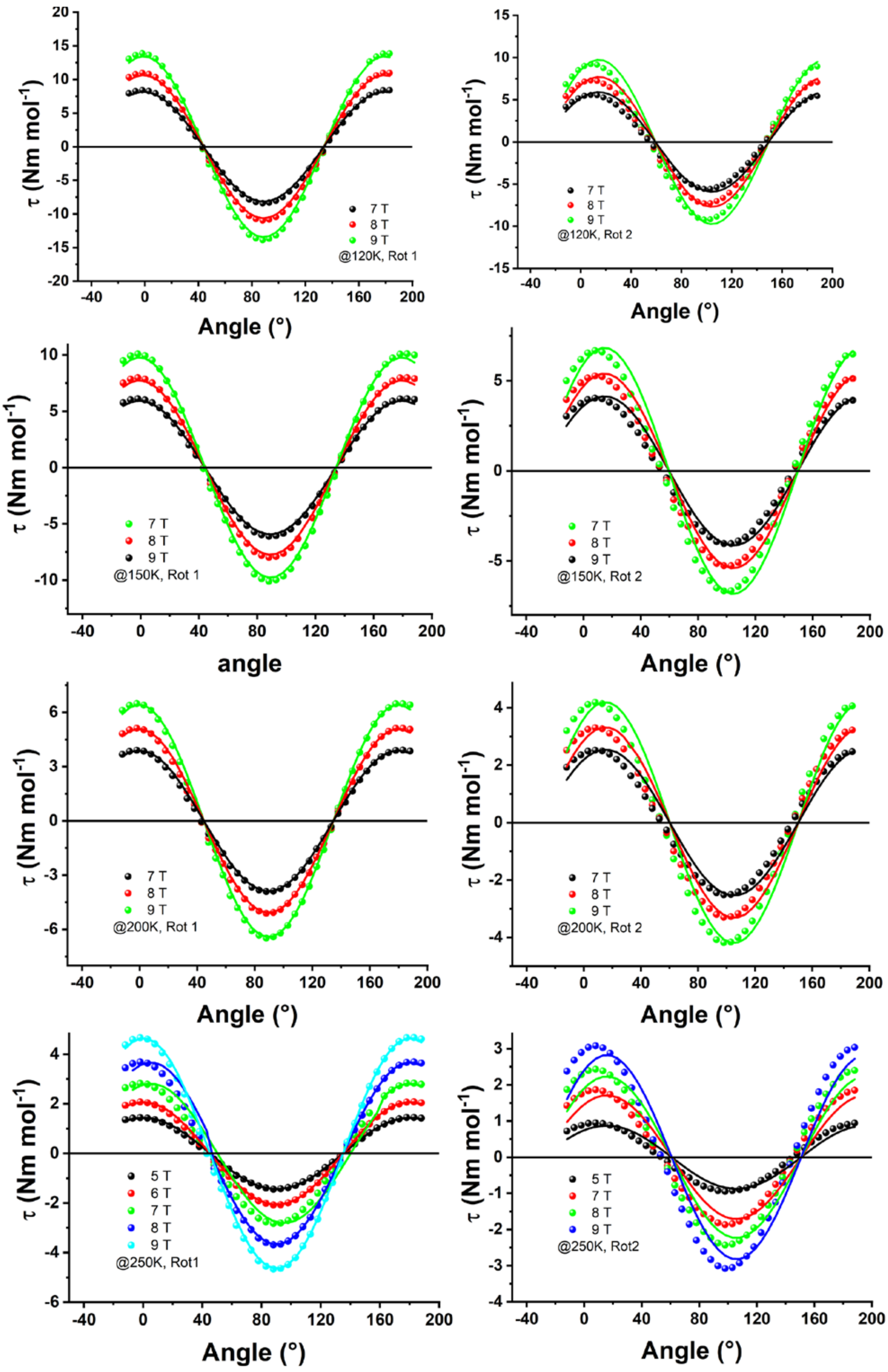

Figure S9: Torque curves obtained at 120-250 K temperatures and at indicated fields for **1**.

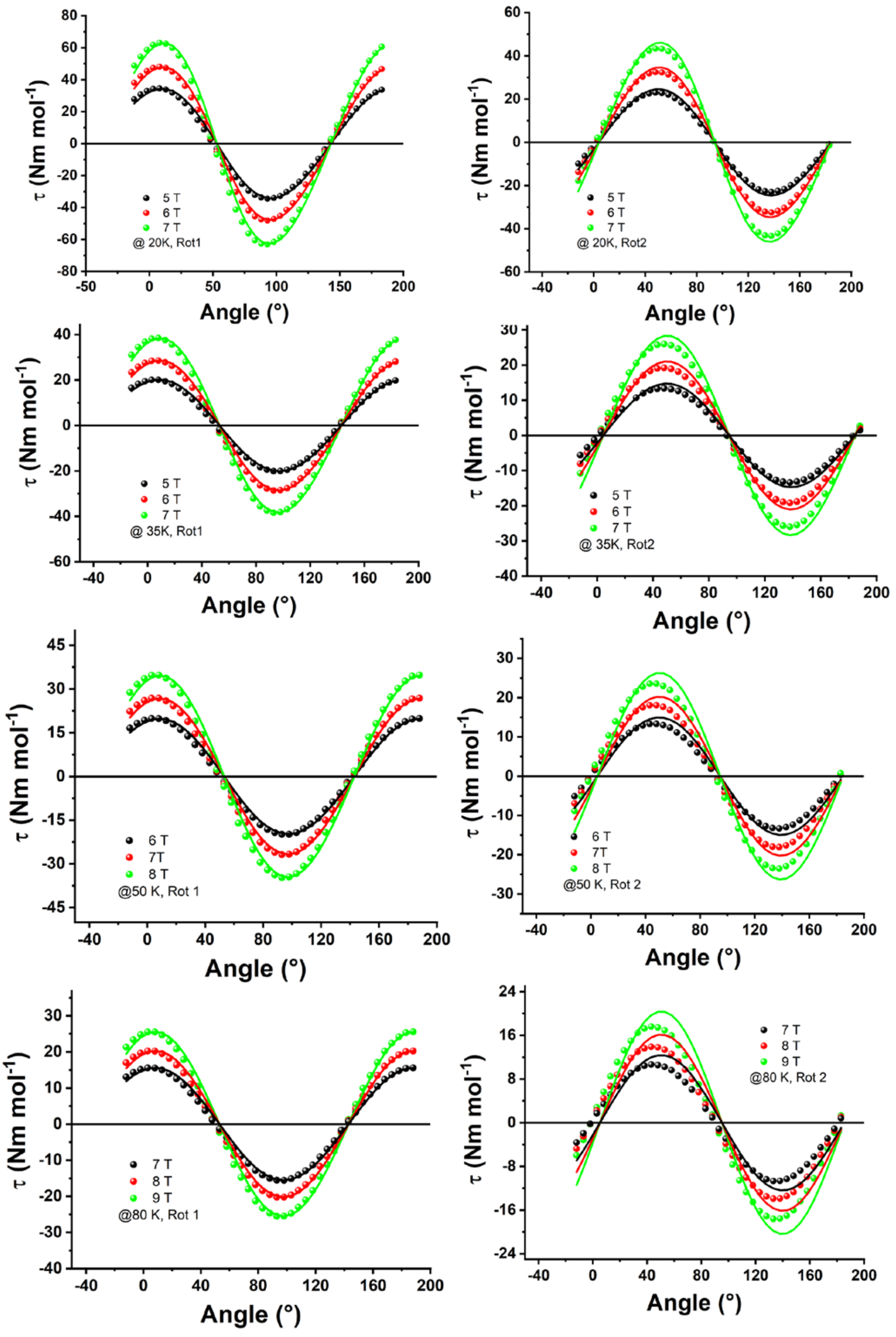

Figure S10: Torque curves obtained at 20-80 K temperatures and at indicated fields for **2**.

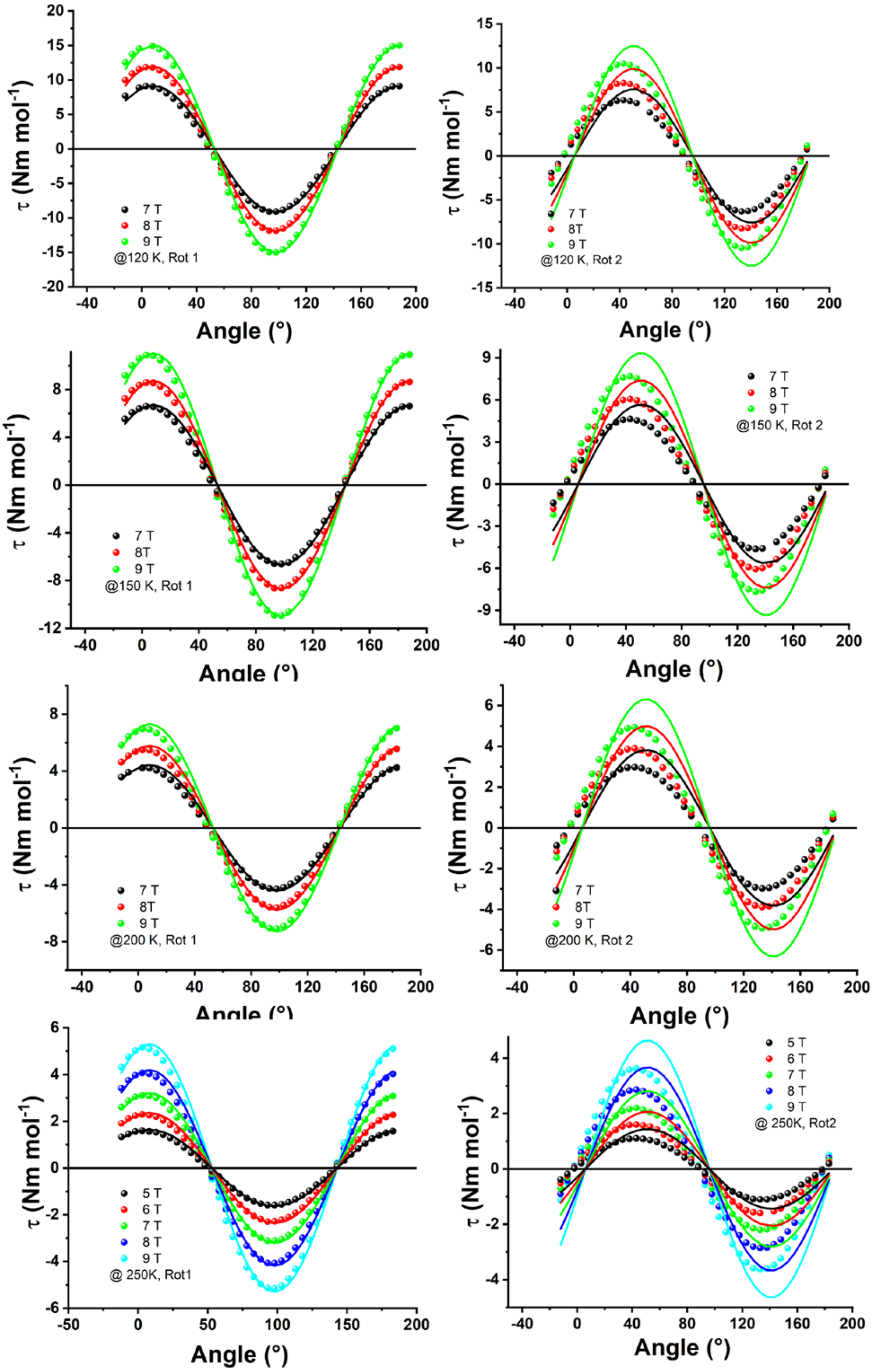

Figure S11: Torque curves obtained at 120-250 K temperatures and at indicated fields for **2**.

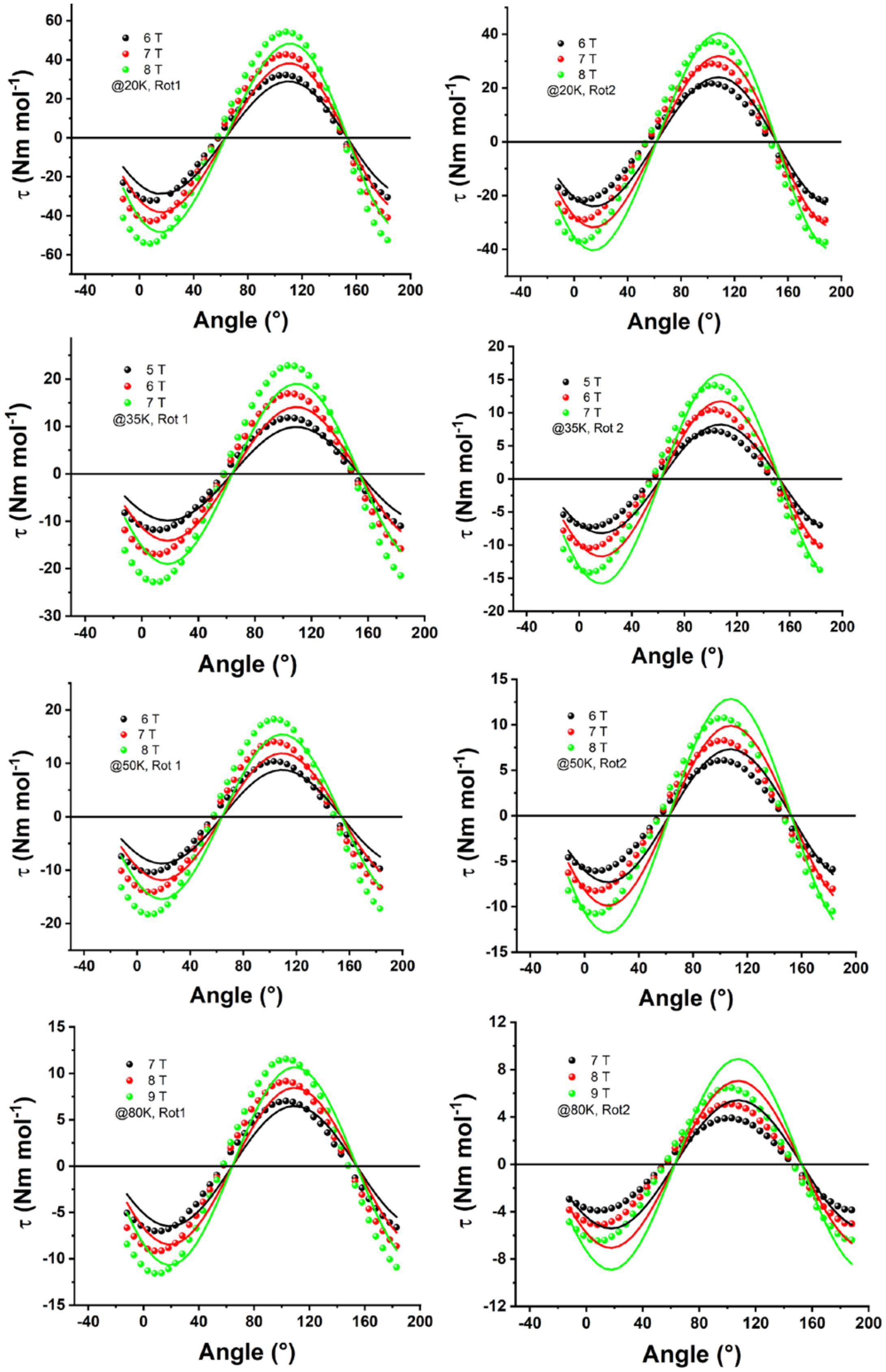

Figure S12: Torque curves obtained at 20-80 K temperatures and at indicated fields for **3**.

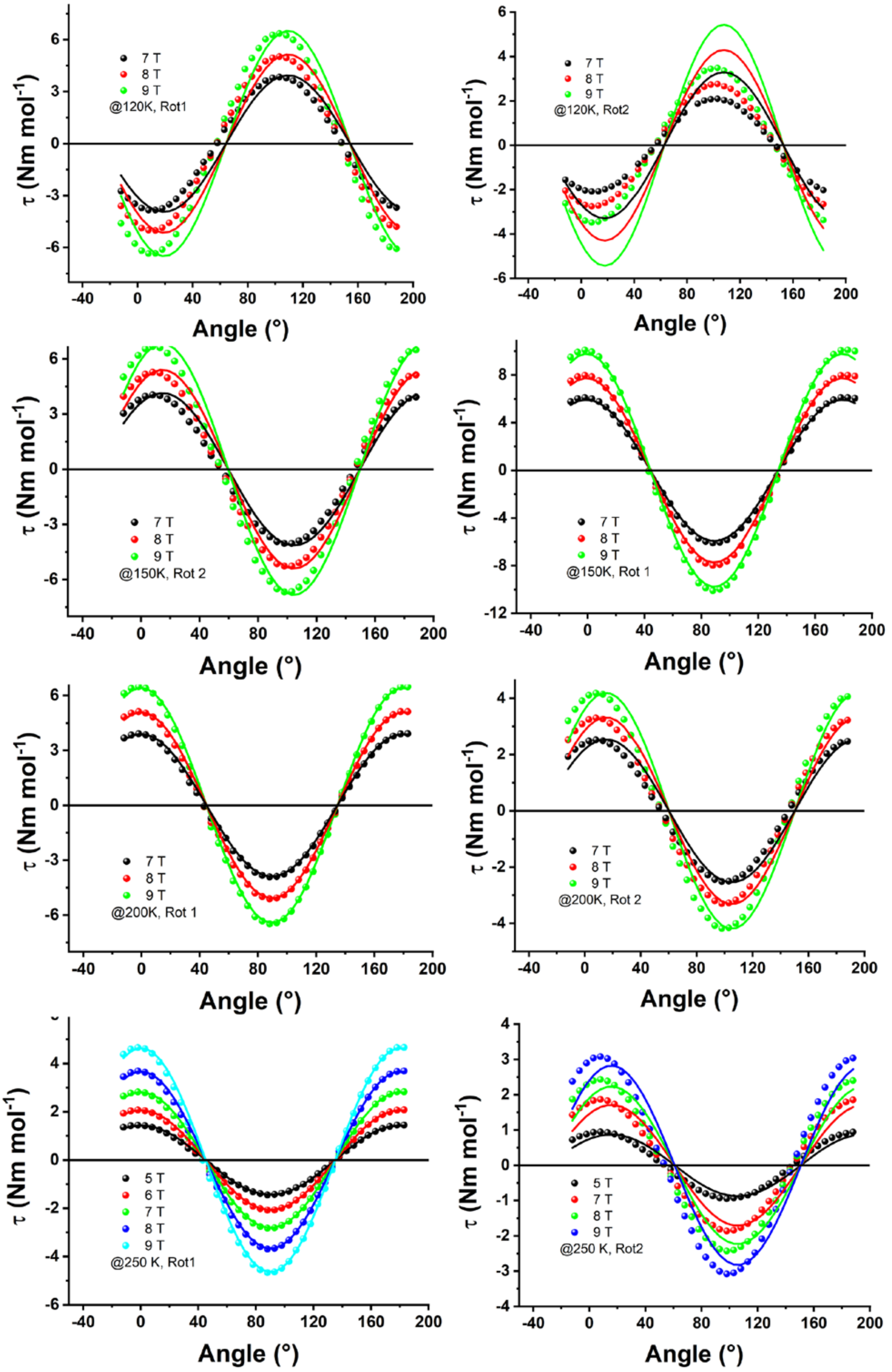

Figure S13: Torque curves obtained at 120-250 K temperatures and at indicated fields for **3**.

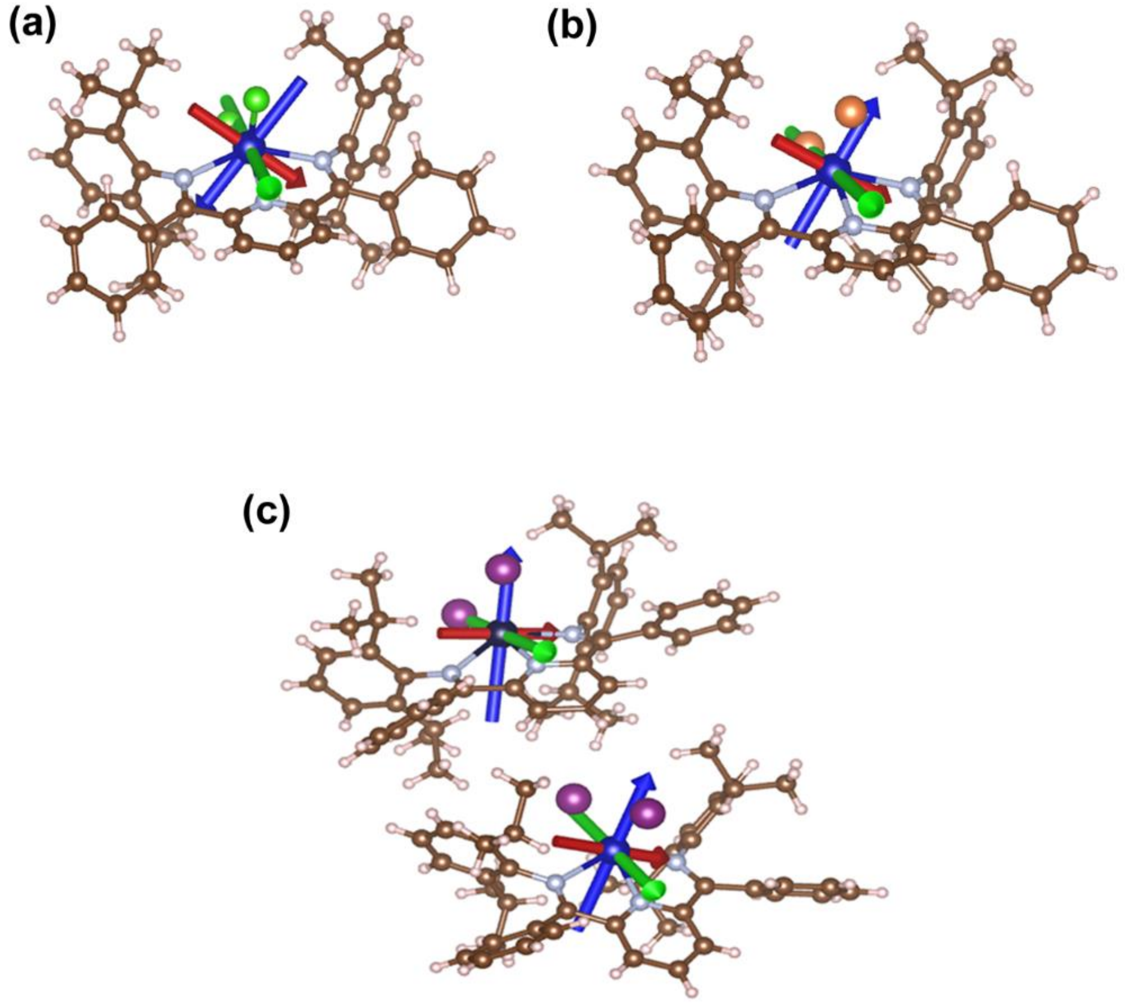

Figure S14: Panel (a-c) are the magnetic anisotropy axes obtained from CTM of **1-3** respectively. Red arrow = g_x, blue arrow = g_y, green arrow = g_z

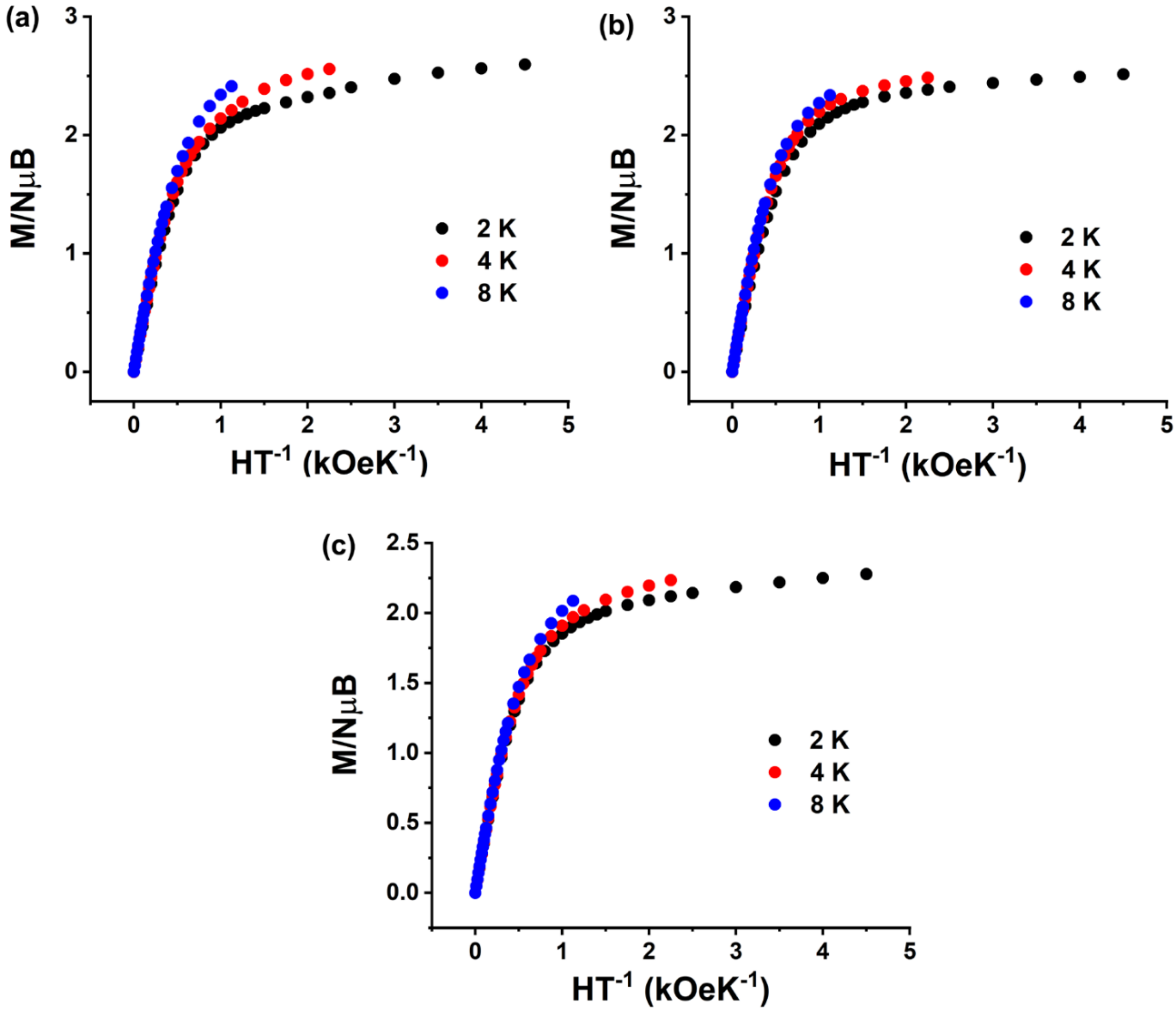

Figure S15: Reduced magnetization data of **1**,**2** and **3** (a-c).

Table S7: NEVPT2 transition energies, their corresponding wavefunctions, and their individual contributions towards D and E values for the ground state and the first five excited states of **1**.

**Spin free energy state of 1**	**Major CASSCF electronic configuration**	**NEVPT2 transition energy (cm^{-1})**	**Contribution to D (cm^{-1})**	**Contribution to E (cm^{-1})**
**GS**	$(d_{xy})^2(d_{yz})^1(d_{xz})^2(d_{z^2})^1(d_{x^2-y^2})^1$ (47%)<br>$(d_{xy})^1(d_{yz})^1(d_{xz})^2(d_{z^2})^1(d_{x^2-y^2})^2$ (13%)<br>$(d_{xy})^1(d_{yz})^2(d_{xz})^2(d_{z^2})^1(d_{x^2-y^2})^1$ (13%)<br>$(d_{xy})^2(d_{yz})^2(d_{xz})^1(d_{z^2})^1(d_{x^2-y^2})^1$ (12%)<br>$(d_{xy})^1(d_{yz})^2(d_{xz})^1(d_{z^2})^1(d_{x^2-y^2})^2$ (10%)	0.0	0.0	0.0
**1st ES**	$(d_{xy})^2(d_{yz})^2(d_{xz})^1(d_{z^2})^1(d_{x^2-y^2})^1$ (47%)<br>$(d_{xy})^2(d_{yz})^1(d_{xz})^1(d_z^{21}(d_{x^2-y^2})^2$ (27%)<br>$(d_{xy})^1(d_{yz})^2(d_{xz})^2(d_{z^2})^1(d_{x^2-y^2})^1$ (17%)	164.4	-137.1	0.006
**2nd ES**	$(d_{xy})^1(d_{yz})^2(d_{xz})^2(d_{z^2})^1(d_{x^2-y^2})^1$ (37%)<br>$(d_{xy})^2(d_{yz})^1(d_{xz})^2(d_{z^2})^1(d_{x^2-y^2})^1$ (21%)<br>$(d_{xy})^2(d_{yz})^1(d_{xz})^1(d_{z^2})^2(d_{x^2-y^2})^1$ (14%)	1612.5	20.3	-20.193
**3rd ES**	$(d_{xy})^2(d_{yz})^1(d_{xz})^1(d_{z^2})^1(d_{x^2-y^2})^2$ (41%)<br>$(d_{xy})^2(d_{yz})^2(d_{xz})^1(d_{z^2})^1(d_{x^2-y^2})^1$ (17%)<br>$(d_{xy})^2(d_{yz})^1(d_{xz})^1(d_{z^2})^2(d_{x^2-y^2})^1$ (16%)<br>$(d_{xy})^2(d_{yz})^1(d_{xz})^2(d_{z^2})^1(d_{x^2-y^2})^1$ (11%)	2904.9	2.4	-2.252
**4th ES**	$(d_{xy})^1(d_{yz})^2(d_{xz})^1(d_{z^2})^2(d_{x^2-y^2})^1$ (67%)<br>$(d_{xy})^1(d_{yz})^1(d_{xz})^1(d_{z^2})^2(d_{x^2-y^2})^2$ (24%)	4985.9	1.5	-1.137
**5th ES**	$(d_{xy})^1(d_{yz})^1(d_{xz})^2(d_{z^2})^1(d_{x^2-y^2})^2$ (55%)<br>$(d_{xy})^1(d_{yz})^1(d_{xz})^2(d_{z^2})^2(d_{x^2-y^2})^1$ (19%)<br>$(d_{xy})^1(d_{yz})^2(d_{xz})^2(d_{z^2})^1(d_{x^2-y^2})^1$ (16%)	5676.6	7.3	7.172

Table S8: NEVPT2 transition energies, their corresponding wavefunctions, and their individual contributions towards D and E values for the ground state and the first five excited states of **2**.

**Spin free energy state of 2**	**Major CASSCF electronic configuration**	**NEVPT2 transition energy (cm^{-1})**	**Contribution to D (cm^{-1})**	**Contribution to E (cm^{-1})**
**GS**	$(d_{xy})^2(d_{yz})^2(d_{xz})^1(d_{z^2})^1(d_{x^2-y^2})^1$ (46%)<br>$(d_{xy})^1(d_{yz})^2(d_{xz})^2(d_{z^2})^1(d_{x^2-y^2})^1$ (28%)<br>$(d_{xy})^2(d_{yz})^1(d_{xz})^1(d_{z^2})^1(d_{x^2-y^2})^2$ (10%)	0.0	0.0	0.0
**1st ES**	$(d_{xy})^2(d_{yz})^1(d_{xz})^2(d_{z^2})^1(d_{x^2-y^2})^1$ (71%)<br>$(d_{xy})^1(d_{yz})^2(d_{xz})^1(d_{z^2})^1(d_{x^2-y^2})^2$ (9%)	484.1	-112.0	0.0
**2nd ES**	$(d_{xy})^1(d_{yz})^2(d_{xz})^2(d_{z^2})^1(d_{x^2-y^2})^1$ (49%)<br>$(d_{xy})^2(d_{yz})^1(d_{xz})^2(d_{z^2})^1(d_{x^2-y^2})^1$ (21%)	2037.8	23.1	-23.185

	$(d_{xy})^1(d_{yz})^1d_{xz})^2(d_{z^2})^2(d_{x^2-y^2})^1$ (14%)			
**3rd ES**	$(d_{xy})^1(d_{yz})^2(d_{xz})^1(d_{z^2})^1(d_{x^2-y^2})^2$ (55%)<br>$(d_{xy})^1(d_{yz})^2(d_{xz})^1(d_{z^2})^2(d_{x^2-y^2})^1$ (16%)<br>$(d_{xy})^1(d_{yz})^2(d_{xz})^2(d_{z^2})^1(d_{x^2-y^2})^1$ (11%)	3958.6	2.1	-2.121
**4th ES**	$(d_{xy})^2(d_{yz})^1(d_{xz})^1(d_{z^2})^2(d_{x^2-y^2})^1$ (83%)	6478.7	1.4	-1.334
**5th ES**	$(d_{xy})^1(d_{yz})^1(d_{xz})^2(d_{z^2})^1(d_{x^2-y^2})^2$ (70%)<br>$(d_{xy})^1(d_{yz})^1(d_{xz})^2(d_{z^2})^2(d_{x^2-y^2})^1$ (20%)	7532.1	6.8	6.864

Table S9: NEVPT2 transition energies, their corresponding wavefunctions, and their individual contributions towards D and E values for the ground state and the first five excited states of **3**.

Spin free energy state of 3	Major CASSCF electronic configuration	NEVPT2 transition energy (cm^{-1})	Contribution to D (cm^{-1})	Contribution to E (cm^{-1})
**GS**	$(d_{xy})^2(d_{xz})^2(d_{yz})^1(d_{z^2})^1(d_{x^2-y^2})^1$ (41%)<br>$(d_{xy})^1(d_{xz})^2(d_{yz})^2(d_{z^2})^1(d_{x^2-y^2})^1$ (29%)<br>$(d_{xy})^1(d_{xz})^1(d_{yz})^2(d_{z^2})^1(d_{x^2-y^2})^2$ (16%)<br>$(d_{xy})^2(d_{xz})^1(d_{yz})^2(d_{z^2})^1(d_{x^2-y^2})^2$ (10%)	0.0	0.0	0.0
**1st ES**	$(d_{xy})^2(d_{xz})^1(d_{yz})^2(d_{z^2})^1(d_{x^2-y^2})^1$ (59%)<br>$(d_{xy})^1(d_{xz})^2(d_{yz})^1(d_{z^2})^1(d_{x^2-y^2})^2$ (28%)	1991.2	-51.9	0.123
**2nd ES**	$(d_{xy})^2(d_x)_z{}^2(d_{yz})^1(d_{z^2})^1(d_{x^2-y^2})^1$ (25%)<br>$(d_{xy})^1(d_{xz})^2(d_{yz})^2(d_{z^2})^1(d_{x^2-y^2})^1$ (21%)<br>$(d_{xy})^2(d_{xz})^1(d_{yz})^2(d_{z^2})^1(d_{x^2-y^2})^1$ (17%)<br>$(d_{xy})^1(d_{xz})^2(d_{yz})^1(d_{z^2})^1(d_{x^2-y^2})^2$ (11%)	3496.4	11.5	-11.5
**3rd ES**	$(d_{xy})^1(d_{xz})^1(d_{yz})^2(d_{z^2})^1(d_{x^2-y^2})^2$ (38%)<br>$(d_{xy})^1(d_{xz})^2(d_{yz})^2(d_{z^2})^1(d_{x^2-y^2})^1$ (16%)<br>$(d_{xy})^1(d_{xz})^2(d_{yz})^1(d_{z^2})^1(d_{x^2-y^2})^2$ (14%)<br>$(d_{xy})^1(d_{xz})^1(d_{yz})^2(d_{z^2})^2(d_{x^2-y^2})^1$ (10%)	4426.7	2.0	-2.046
**4th ES**	$(d_{xy})^1(d_{xz})^2(d_{yz})^1(d_{z^2})^1(d_{x^2-y^2})^2$ (30%)<br>$(d_{xy})^1(d_{xz})^1(d_{yz})^2(d_{z^2})^1(d_{x^2-y^2})^2$ (29%)<br>$(d_{xy})^2(d_{xz})^2(d_{yz})^1(d_{z^2})^1(d_{x^2-y^2})^1$ (18%)<br>$(d_{xy})^1(d_{xz})^2(d_{yz})^1(d_{z^2})^2(d_{x^2-y^2})^1$ (14%)	6461.6	6.4	6.942
**5th ES**	$(d_{xy})^2(d_{xz})^1(d_{yz})^1(d_{z^2})^2(d_{x^2-y^2})^1$ (79%)<br>$(d_{xy})^1(d_{xz})^1(d_{yz})^1(d_{z^2})^2(d_{x^2-y^2})^2$ (13%)	9489.2	0.6	-0.629

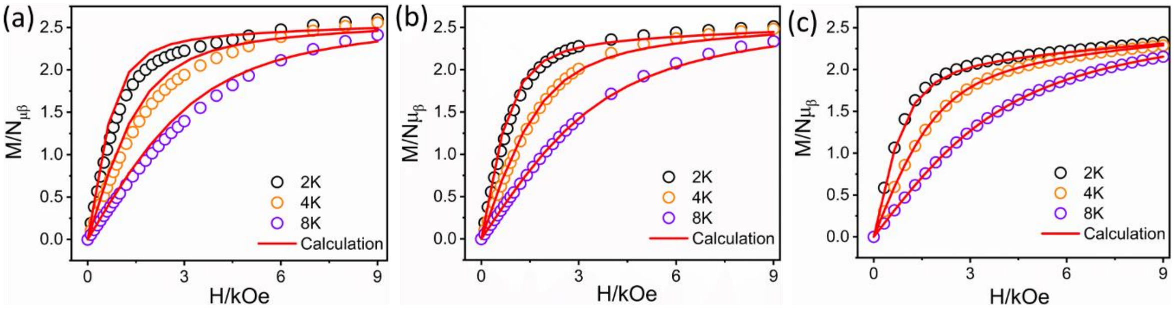

Figure S16: (a-c) Comparison of observed isothermal field-dependent magnetization measurements with NEVPT2 calculations for **1-3** respectively. Experimental magnetization at 2 K (red), 4 K (orange), and 8 K (violet) is represented by symbols, while the red solid line at each temperature shows the calculated magnetization from NEVPT2.

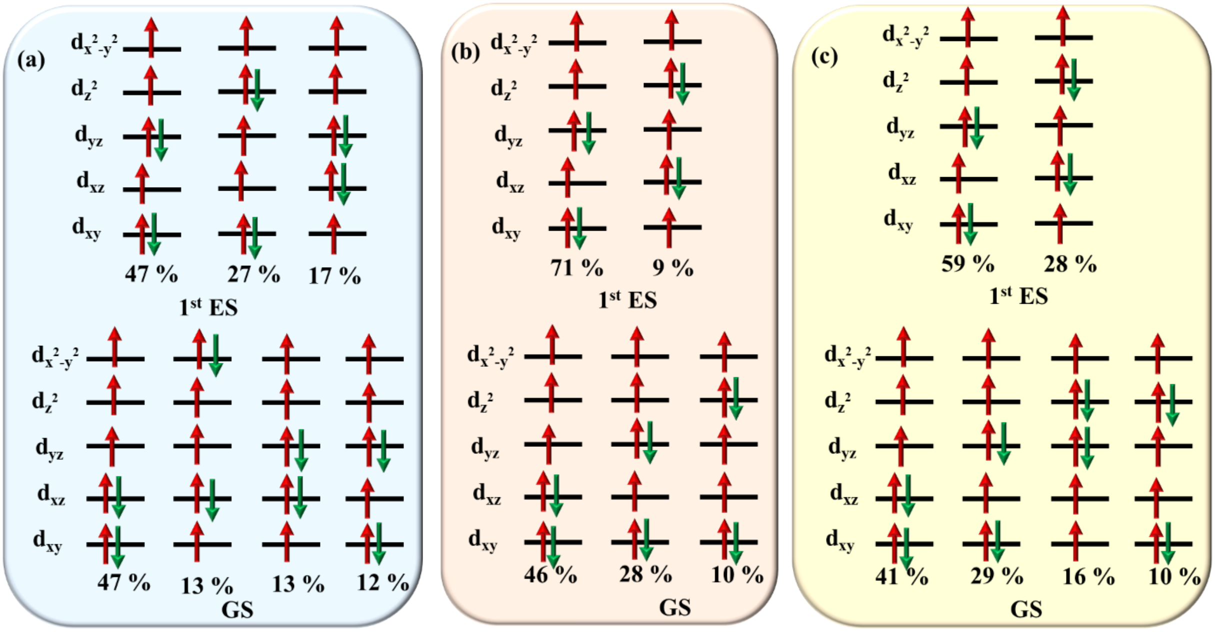

Figure S17: (a-c) Composition of the wavefunctions corresponding to the ground state (bottom) and the first excited state (top), including only the determinants with the major contributions of **1-3** respectively.

Table S10: Ligand field parameters derived from AILFT calculations conducted at the NEVPT2 level of theory for **1**, **2**, and **3**. The values of the B, C, and ξ parameters are given in units of cm^{-1}.

**Parameter**	**Free Co (II)**	**1**	**2**	**3**	**% reduction**		
					**1**	**2**	**3**
ζ	527.0	512.0	503.5	490.8	2.84	4.45	6.86
B	1039.8	999.4	1000.7	1003.4	3.88	3.76	3.500
C	4160.3	3913.9	3900.7	3874.8	5.92	6.23	6.86
C/B	4.001	3.92	3.90	3.86	2.02	2.52	3.52

Table S11: All the optimized model complex structures, their distances from the basal plane (in Å), and their SH parameters.

Model	Optimized Structure	Distance from the basal plan (Å)	D (cm^{-1})	E/D	g_{xx}, g_{yy}, g_{zz}
**1-H_2O**	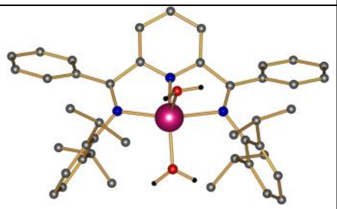	0.515	-35.7	0.161	2.131, 2.271, 2.597
**1-F**	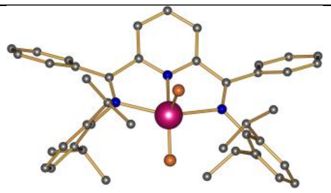	0.678	-60.3	0.203	2.004, 2.234, 2.765
**1-OH**	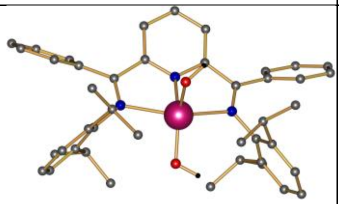	0.697	-66.6	0.152	2.000, 2.179, 2.810

**1-Me**	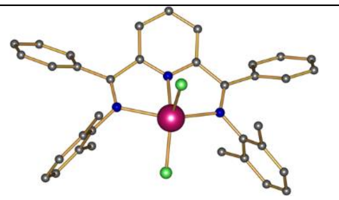	0.716	-122.1	0.117	1.791, 2.021, 3.355
**1-Et**	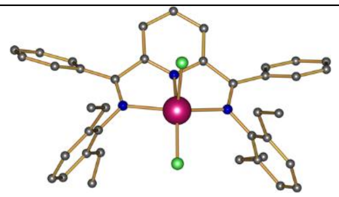	0.577	-75.82	0.171	2.094, 2.331, 2.868
**1-Si(Me)$_3$**	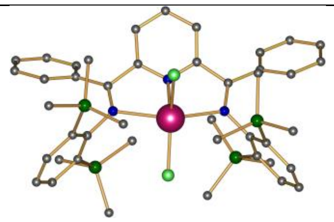	0.709	-139.9	0.134	1.341, 1.582, 3.478
**1-^{t}Bu**	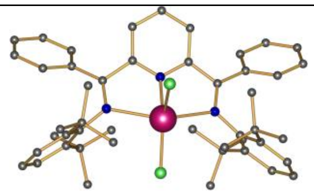	0.724	-145.9	0.099	1.436, 1.682, 3.538
**1-^{t}(Bu)$_3$**	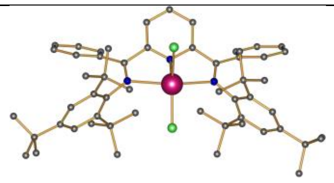	0.718	-144.8	0.102	1.446, 1.694,3.529

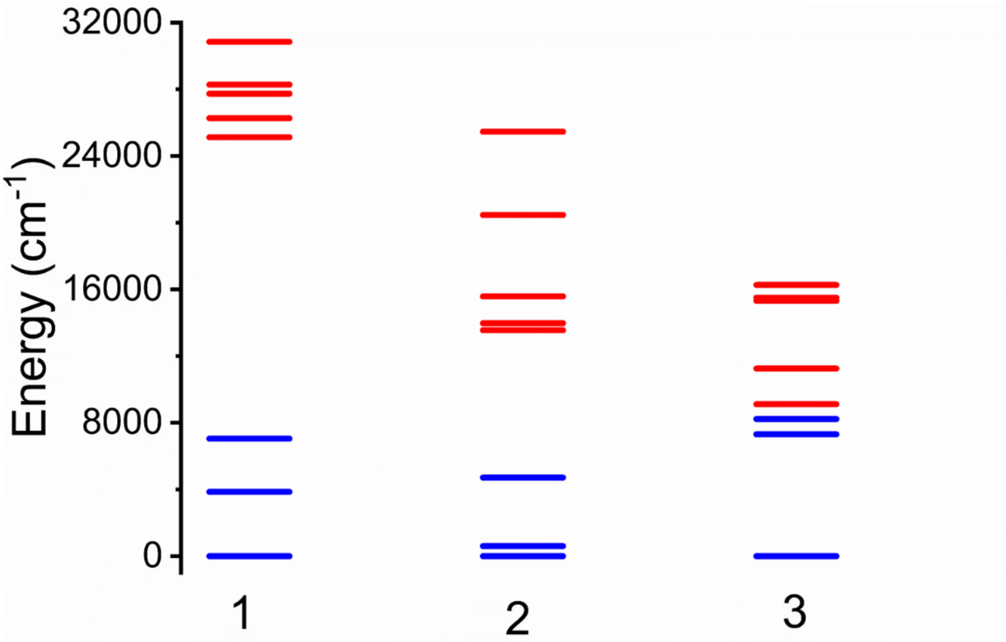

Figure S18: CASSCF computed orbital energies for **1**–**3**. The thick blue lines indicate the σ-bonding orbitals, while the red lines represent the d-orbital splitting.

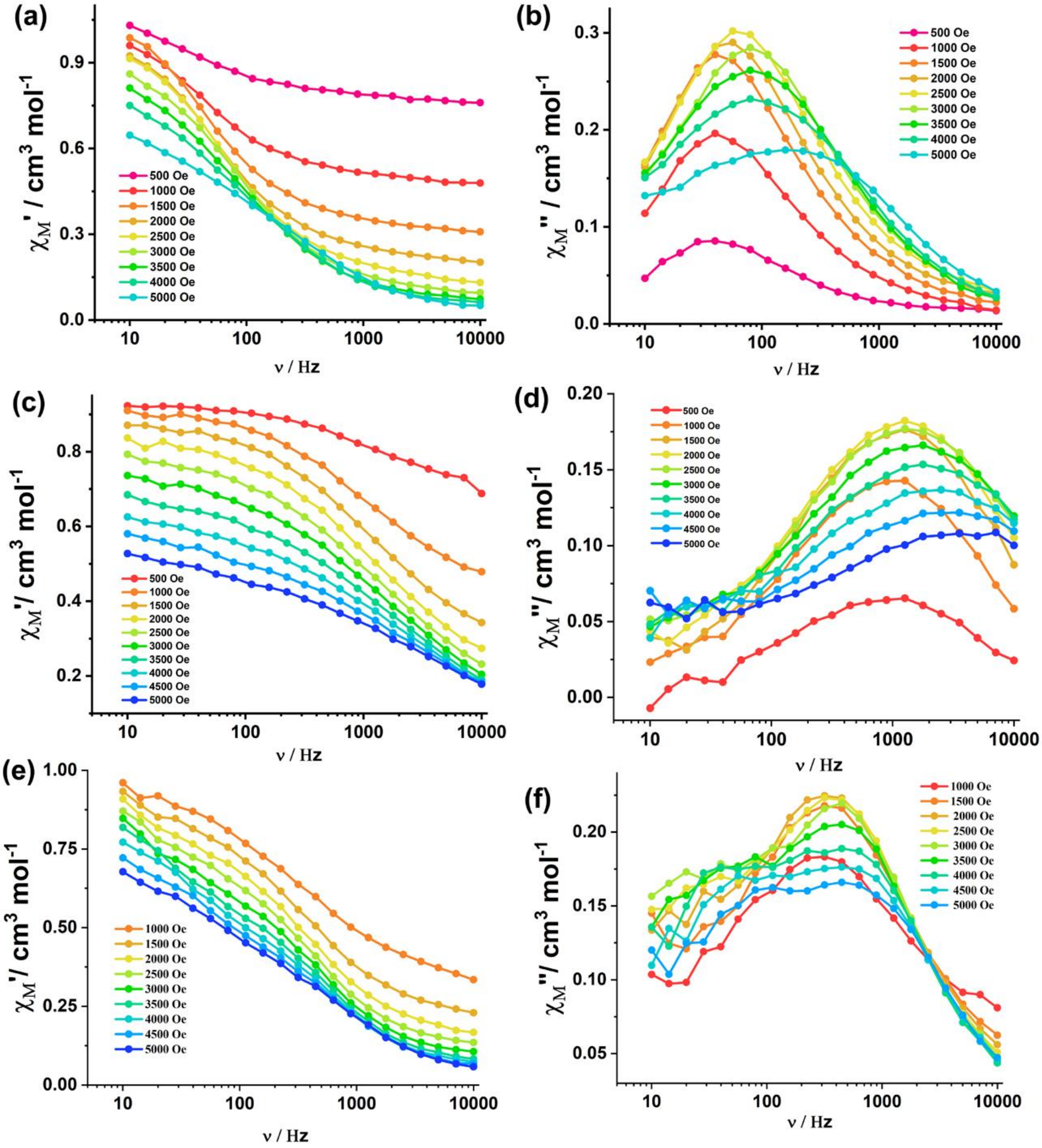

Figure S19: Field-dependent in-phase and out-of-phase susceptibility signals of **1**(a,b), **2(**c,d) and **3**(e,f) at 2 K.

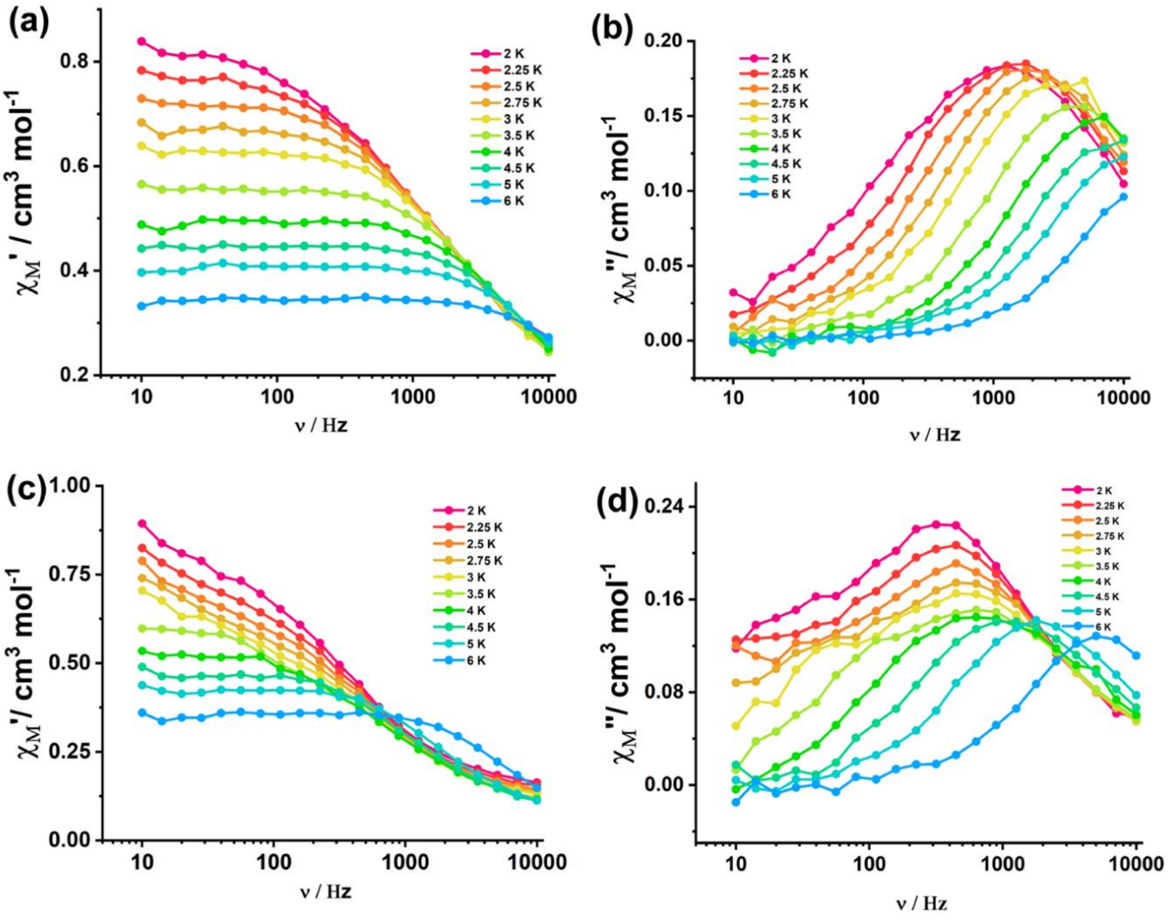

Figure S20: Frequency-dependent in-phase and out-of-phase susceptibility signals of **2**(a,b) and **3(**c,d) at 2 kOe DC field.

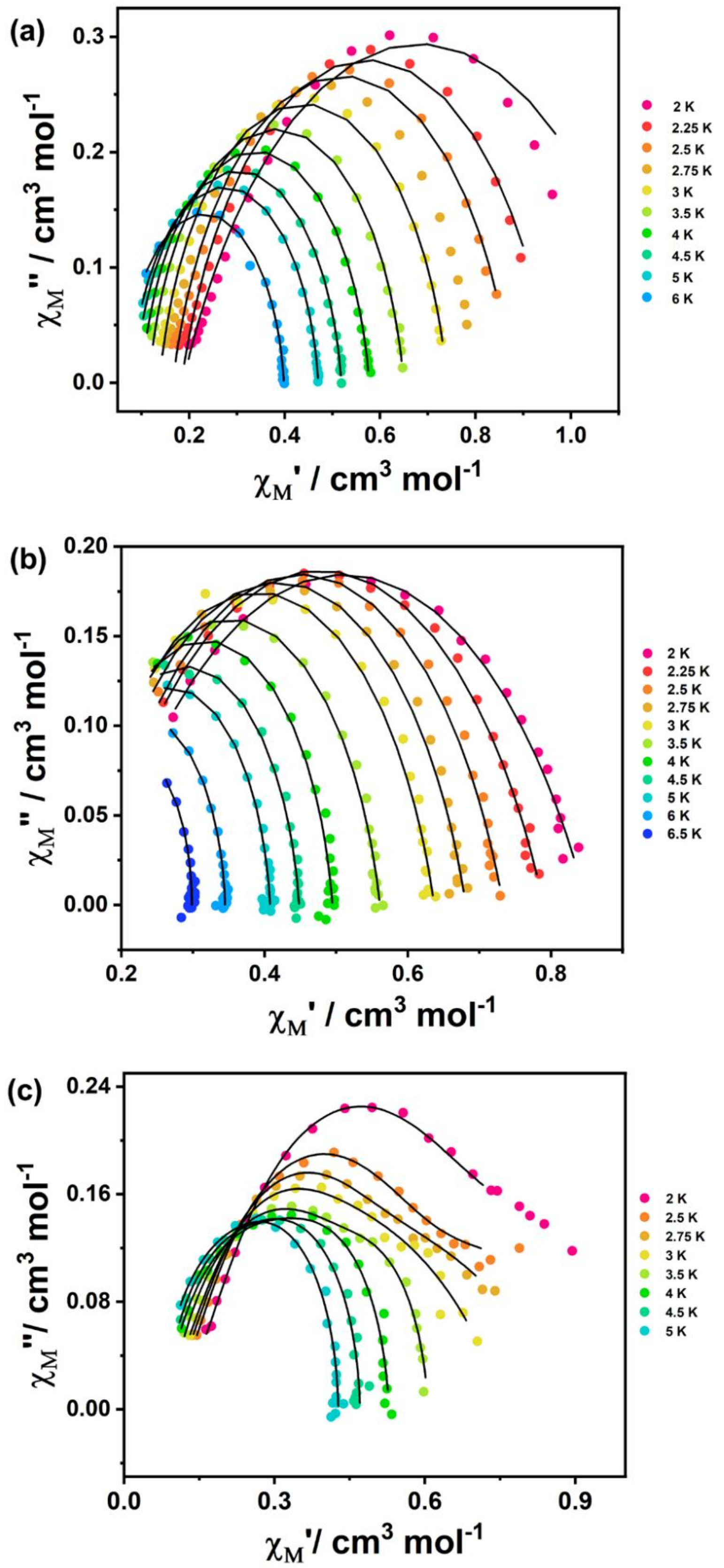

Figure S21: Cole-Cole plot at the indicated temperatures of complexes **1-3** (a-c)

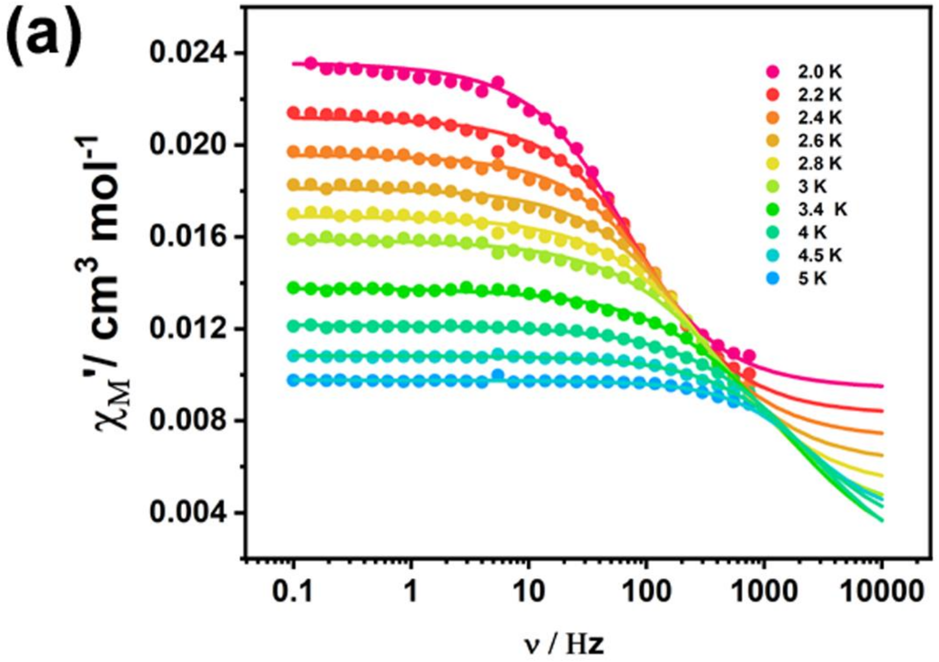

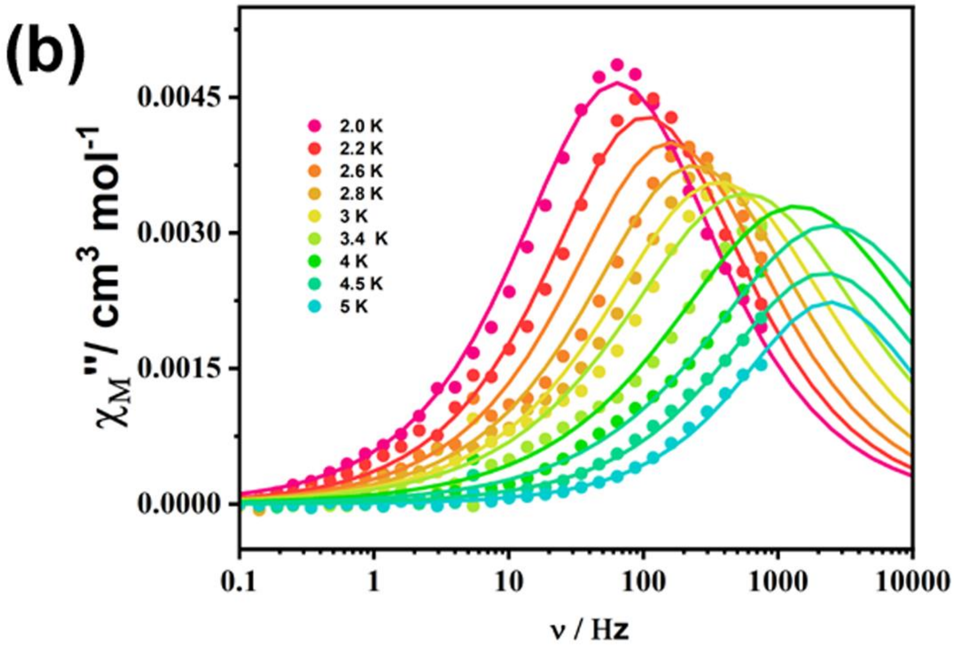

Figure S22: (a,b) **2-dil** at 0.06 T applied DC field. The solid lines are the best fit using the Debye model. The solid lines have been extended up to 10,000 Hz using the fitting parameters obtained from experimental AC data.

Table S12: Fitting parameters for Cole-Cole plot for complex **1**.

**S.No.**	**Temp. (K)**	χ_S	χ_T	**τ**	**α**
1	2	0.18659	1.17617	0.00371	0.31768
2	2.25	0.18376	0.96211	0.0019	0.20613
3	2.5	0.16512	0.87587	0.00129	0.18162
4	2.75	0.14968	0.80318	8.95216E-4	0.15883
5	3	0.13716	0.74105	6.4242E-4	0.14013
6	3.5	0.11516	0.64934	3.6308E-4	0.12247
7	4	0.09993	0.57691	2.22358E-4	0.10905
8	4.5	0.08616	0.5187	1.43788E-4	0.10274
9	5	0.0775	0.47027	9.80196E-5	0.09251
10	6	0.06293	0.39819	4.96522E-5	0.0845

Table S13: Fitting parameters for Cole-Cole plot for complex **2**.

**S.No.**	**Temp. (K)**	χ_S	χ_T	**τ**	**α**
1	2	0.17026	0.8505	1.34514E-4	0.36776
2	2.25	0.16573	0.78971	1.06052E-4	0.31332
3	2.5	0.16756	0.73296	8.67582E-5	0.26377
4	2.75	0.15662	0.68099	6.85046E-5	0.23341
5	3	0.14355	0.63676	5.40325E-5	0.21576
6	3.5	0.14181	0.56099	3.86758E-5	0.17124
7	4	0.13966	0.49468	2.82252E-5	0.11693
8	4.5	0.13399	0.44807	2.1611E-5	0.105473
9	5	0.13073	0.40753	1.72202E-5	0.0833
10	6	0.11985	0.34475	1.08622E-5	0.05153

Table S14: Fitting parameters for Cole-Cole plot for complex **3.**

**S.No.**	**Temp. (K)**	χ_S	χ_T	τ	α
1	2	0.09212	0.96209	6.79771E-4	0.41745
2	2.25	0.07386	0.89384	5.99902E-4	0.43909
3	2.5	0.05825	0.84199	5.38579E-4	0.45693
4	2.75	0.05212	0.79652	4.88313E-4	0.46016
5	3	0.0593	0.73375	4.24965E-4	0.43249
6	3.5	0.07173	0.63017	3.04316E-4	0.36008
7	4	0.08285	0.54165	2.01855E-4	0.26572
8	4.5	0.08452	0.47771	1.31043E-4	0.18643
9	5	0.08346	0.42878	8.25177E-5	0.12032

References

1. Jurca, T.; Dawson, K.; Mallov, I.; Burchell, T.; Yap, G. P.; Richeson, D. S., Disproportionation and radical formation in the coordination of "GaI" with bis(imino)pyridines. *Dalton Trans.* **2010,** *39* (5), 1266-72.
2. Perfetti, M., Cantilever torque magnetometry on coordination compounds: from theory to experiments. *Coord. Chem. Rev.* **2017,** *348*, 171-186.
3. Sarkar, A.; Tewary, S.; Sinkar, S.; Rajaraman, G., Magnetic Anisotropy in CoIIX4 (X= O, S, Se) Single-Ion Magnets: Role of Structural Distortions versus Heavy Atom Effect. *Chem. Asian J.* **2019,** *14* (24), 4696-4704.
4. Acharya, J.; Sarkar, A.; Kumar, P.; Kumar, V.; Flores Gonzalez, J.; Cador, O.; Pointillart, F.; Rajaraman, G.; Chandrasekhar, V., Influence of ligand field on magnetic anisotropy in a family of pentacoordinate CoII complexes. *Dalton Trans.* **2020,** *49* (15), 4785-4796.
5. Sarkar, A.; Dey, S.; Rajaraman, G., Role of coordination number and geometry in controlling the magnetic anisotropy in FeII, CoII, and NiII single-ion magnets. *Chem. Eur. J.* **2020,** *26* (62), 14036-14058.
